# Microenvironment-derived acetylated amino acids promote glioblastoma treatment resistance

**DOI:** 10.21203/rs.3.rs-10976328/v1

**Published:** 2026-09-18

**Authors:** Palavalasa Sravya, Leyla Nurcihan Altay, Kathy Do, Jack Freeman, Aditri Gokul, Ningning Liang, Andrew J Scott, Jie Xu, Navyateja Korimerla, Angelica Lin, Heng Wang, Kari Wilder-Romans, Ava Singer, Alexandra O’Brien, Olamide Animasahun, Baharan Meghdadi, Emily Baker, Erik R Peterson, Elizabeth C McCulla, Ziqing Zhu, Dafydd Thomas, Anthony Andren, Harrison Wong, Peter Sajjakulnukit, Li Zhang, Sunita Shankar, Visweswaran Ravikumar, Zheng Hong Lee, Joseph A. Nieto Carrion, Sagnik Bhadury, C. Ryan Miller, Jann N. Sarkaria, Jason A. Heth, Jason T Huse, Sandra Camelo-Piragua, Meredith A Morgan, Theodore S Lawrence, Arvind Rao, Costas Lyssiotis, Wajd Al-Holou, Deepak Nagrath, Weihua Zhou, Daniel R. Wahl

**Affiliations:** 1. Department of Radiation Oncology, University of Michigan, Ann Arbor, MI, USA; 2. Department of Chemical engineering, University of Michigan, Ann Arbor, MI, USA; 3. Department of Pathology, University of Michigan, Ann Arbor, MI, USA; 4. Department of Molecular & Integrative Physiology, University of Michigan, Ann Arbor, MI, USA; 5. Department of Computational Medicine and Bioinformatics, University of Michigan, Ann Arbor, MI, USA; 6. Department of Neurosurgery, University of Michigan, Ann Arbor, MI, USA; 7. Department of Pathology, The University of Texas MD Anderson Cancer Center, Houston, TX, USA; 8. Department of Translational Molecular Pathology, The University of Texas MD Anderson Cancer Center, Houston, TX, USA.; 9. Department of Biomedical Engineering, University of Michigan, Ann Arbor, MI, USA; 10. Rogel Cancer Center, University of Michigan, Ann Arbor, MI, USA; 11. Department of Internal Medicine, Division of Gastroenterology, University of Michigan, Ann Arbor, MI, USA; 12. Michigan Center for Translational Pathology, University of Michigan, Ann Arbor, MI, USA; 13. Laboratory for Systems Biology of Human Diseases, University of Michigan, Ann Arbor, MI, USA.; 14. Biointerfaces Institute, University of Michigan, Ann Arbor, MI, USA; 15. Department of Pathology, University of Alabama Birmingham, Birmingham, AL, USA; 16. Department of Radiation Oncology, Mayo Clinic, Rochester, MN, USA

## Abstract

Rapid repair of genotoxic therapy induced DNA damage mediates treatment resistance in glioblastoma (GBM). The role of non-malignant cell types in the tumor microenvironment in accelerating DNA repair in neoplastic cells is poorly understood. Using spatial transcriptomics, immunofluorescence, metabolomics, patient tissues and preclinical models, we show that tumor associated macrophages (TAMs) in GBM promote DNA repair and treatment resistance in neoplastic cells through the secretion of acetylated amino acids. These acetylated amino acids are consumed by GBM cells, leading to enhanced acetyl Co-A levels, histone acetylation and nucleotide synthesis. Interrupting histone acetylation via inhibition of the acetyltransferase KAT5 breaks these metabolic links and reverses the protective capacity of microenvironment-derived acetylated amino acids and TAMs. Blocking the exchange of acetylated amino acids and their downstream effects is a potential strategy for the treatment of GBM.

## Introduction

Glioblastoma (GBM) is one of the most aggressive and treatment-resistant forms of adult brain cancer, with a median survival of approximately 16 months despite standard of care which includes radiation therapy (RT) and temozolomide (TMZ)^1^. Both these treatment modalities primarily act by inducing DNA damage. Rapid repair of this damage in neoplastic cells leads to treatment resistance in GBM^2^. Mounting evidence suggests that metabolic alterations in GBM cells influence DNA repair and genotoxic therapy resistance^3–5^, but how and whether microenvironment-derived metabolites are involved is uncertain.

The GBM microenvironment is heterogeneous, with a high proportion of non-malignant cells in the tumor mass that actively contribute to tumor growth and therapy resistance^6–10^. Myeloid cells, including brain-resident microglia and macrophages recruited to the tumor from the periphery, are the most abundant non-malignant cell types in GBM, and subsets are associated with enhanced DNA repair in murine glioblastoma^9^. Furthermore, metabolic cross talk between non-malignant and malignant cells drives responses to treatment in various cancer types^11–13^. Taken together, these data suggest that the non-malignant cells in the glioblastoma tumor microenvironment may play a role in glioblastoma treatment resistance by promoting DNA repair and rewiring metabolism. However, the nature of metabolic cross talk between GBM cells with their neighbors and whether any such communication regulates DNA repair and treatment resistance in GBM is unknown.

Here, we investigate how the microenvironment influences the repair of radiation-induced DNA damage in GBM cells in mouse models and human GBM tissues through spatial transcriptomics coupled with immunofluorescence (IF). Our findings across patient tissues and model systems indicate that proximity to myeloid cells is a key factor associated with rapid DNA repair in neoplastic glioma cells across models and patients. We further show that this phenotype is mediated through the transfer of N-acetylated amino acids from myeloid cells to neoplastic cells, which promotes their metabolic activity, histone acetylation, DNA repair and RT resistance. Interrupting these newly discovered metabolic links overcomes microenvironment-driven therapeutic resistance in GBM and could be a new therapeutic strategy for this disease.

## RESULTS

### The intracranial environment promotes GBM DNA repair and RT resistance

**Fig1.**
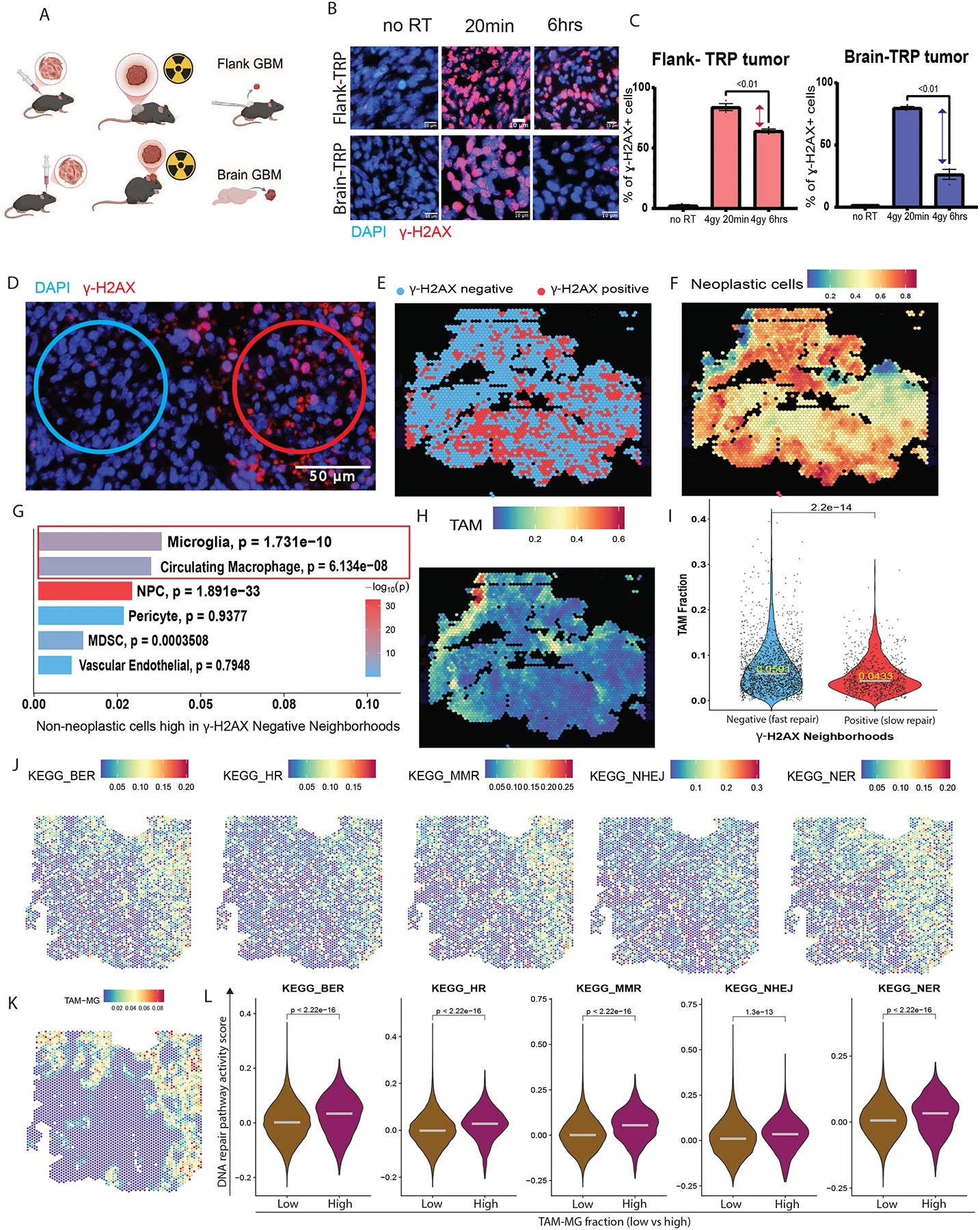
The brain microenvironment assists rapid spatially-heterogeneous DNA repair in glioblastoma. **A.** Illustration depicting experimental design for generating TRP tumors in the flank and the brain of immunocompetent mice. **B.** Representative images of γ-H2AX immunofluorescence in flank tumors and brain tumors after radiation therapy. Scale bar:10μm. **C.** Quantification of % γ-H2AX^+^ cells at 20min and 6hrs following 4 Gy RT. The larger magnitude of the double-sided blue arrow (intracranial) than the double-sided red arrow (flank) suggests enhanced resolution of DNA damage at 6hrs in the intracranial tumor (n=3 tumors for each condition). P-value calculated using unpaired T-test. **D.** Representative image showing spatial heterogeneity in γ-H2AX immunofluorescence 6hrs after irradiation in a TRP tumor in mouse brain (Blue circle showing γ-H2AX negative region and red circle showing γ-H2AX positive region). **E.** Spatial map showing distinct regions of γ-H2AX positive (slow repair) and γ-H2AX negative (fast repair) 6hrs after RT. **F.** Spatial map of neoplastic cell distribution. **G.** Graph showing mean fractional abundance of non-malignant cell types that are overrepresented in the γ-H2AX negative neighborhoods. P-values were calculated using Wilcoxon test for comparison of fractional abundance of each cell type in γ-H2AX negative and γ-H2AX positive neighborhoods. **H.** Spatial map showing distribution of tumor associated macrophages (TAMs). **I.** Fractional abundance of TAMs in γ-H2AXpositive and negative neighborhoods. P-value calculated using Wilcoxon test. **J, K.** Spatial maps of a representative human glioblastoma tissue showing DNA repair pathway activity distribution and microglial (TAM-MG) distribution. **L.** Transcriptional DNA repair activity in regions with microglia high and low abundance, in 8 unirradiated human glioblastoma tissue sections obtained from 3 patients. P-values calculated using Wilcoxon test.

From our prior studies^3,14^, we observed that GBM tumors (patient-derived models GBM6, HF2303, GBM38) grown orthotopically in mouse brain showed more resistance to radiation therapy (RT) than when grown in mouse flanks^3,14^. When grown in mouse flanks, all 3 PDX models responded to RT (2 Gy × 4–5 fractions) with dramatically slower growth (FigS1 A–C) and took significantly longer to reach end point (tumor tripling, Fig S1 D–F). By contrast, the same models grown orthotopically in mouse brains, showed minimal response to same or higher dose of RT (2 Gy × 5–10 fractions) with minimal to no difference in rate of tumor growth (Fig1S G–I) as well as in the time to reach the end point (survival, Fig1S J–L). While there are numerous possible explanations for these differences, we hypothesized that location-specific differential RT responses might be governed by microenvironment-driven regulation of DNA repair, since RT primarily exerts its efficacy through unrepaired double strand DNA breaks.

We thus tested whether the observed RT resistance in intracranial tumors was associated with poorer induction of DNA damage or enhanced DNA repair. We assessed this by comparing RT responses in tumors derived from syngeneic murine TRP GBM cells^15^ grown in immunocompetent mouse brain or flanks (Fig1A). We first used immunofluorescence to assess the formation and resolution of RT-induced γ-H2AX foci, a variant histone that is phosphorylated by apical DNA damage sensing kinases, serves as a scaffold for DNA repair protein recruitment, and then is dephosphorylated over time as DNA damage is repaired^16–18^. We also assessed the formation and resolution of foci of 53BP1, a protein recruited to sites of DNA damage to assist in the repair of double strand breaks that delocalizes once DNA damage is repaired^19,20^. GBM tumors in the flank and brain have similar, uniformly high positivity for 53BP1 and γ-H2AX foci within minutes of RT (Fig 1B, 1C and Fig S1M, S1N), suggesting similar induction of DNA damage in both intracranial and flank tumors. At 6hrs after RT, as expected, significantly fewer cells were positive for 53BP1 and γ-H2AX foci in both flank and the brain derived tumors (Fig 1B,1C and Fig S1M, S1N). However, residual staining of DNA damage markers at 6hrs after RT is significantly lower in brain compared to flank tumors (Fig 1B,1C and Fig S1M and S1N). The more rapid resolution of RT-induced DNA damage markers and impaired ability of RT to slow the growth of intracranial tumors suggest that the brain environment might promote RT resistance by enhancing DNA repair.

### Spatial heterogeneity in GBM DNA repair is associated with tumor associated macrophage (TAM) proximity

We then more closely examined the patterns of fluorescent marker resolution following RT in intracranial tumors. Focusing on γ-H2AX (as its formation and resolution are broadly applicable to multiple DNA repair pathways^17^), we observed striking evidence of spatially distinct tumor staining 6 hr following RT (Fig 1D). Because of the nearly uniform positive γ-H2AX staining immediately after RT (Fig 1C), these findings suggest the existence of spatially distinct tumor neighborhoods with rapid DNA repair (γ-H2AX negative) and with slower DNA repair (γ-H2AX positive). This spatial heterogeneity in γ-H2AX resolution following RT suggests that GBM DNA repair may be influenced by its heterogenous microenvironment.

To identify potential regulators of the observed spatial heterogeneity in DNA repair, we combined γ-H2AX immunofluorescence with spatial transcriptomics in irradiated and control orthotopic syngeneic GBM tumors. Our initial efforts used “spot-based” spatial transcriptomics (the leading technology available at its time) coupled with γ-H2AX immunofluorescence performed on the same slide (Visium CytAssit CG000495). Since the spatial resolution of this spot-based platform is 55 um, rather than single cells, we defined each spot as a neighborhood. We then annotated these neighborhoods as γ-H2AX positive (suggestive of slow repair of DNA damage) or γ-H2AX negative (suggestive of fast repair of damage) using a MATLAB tool we previously described^21^. Briefly, neighborhoods whose cellular contents stained at 10% or more positive for γ-H2AX were annotated as γ-H2AX positive (Fig 1E). We then deconvoluted the cellular composition of the neighborhoods using single cell RNA sequencing data obtained from unirradiated TRP tumors from the brain, as the reference dataset (Figs S1O,P). To maintain our focus on neoplastic cell DNA repair, we restricted our analysis to neighborhoods comprised of at least 40% neoplastic cells (Fig 1F, Figs S1Q,R). By comparing the non-malignant cell composition of the neoplastic rich neighborhoods with fast repair (γ-H2AX negative) and slow repair (γ-H2AX positive), we identified microglia and macrophages as being most strongly associated with fast repair (Fig1 G–H). Microglia are the resident macrophages of the brain and together with recruited circulating macrophages, comprise a population of cells termed tumor associated macrophages (TAMs)^22^. When analyzed together, TAM content is elevated in neoplastic-rich spots with rapid resolution of γ-H2AX(Fig 1I).

We next sought to examine these relationships in human GBM tissue. In current GBM treatment paradigms, surgical resection of tumors typically occurs before or long after genotoxic treatments, which limits the ability to study DNA repair in patients. We therefore assessed the spatial distribution and expression of genes involved in DNA repair in resected unirradiated GBM tumors as a surrogate for tumor DNA repair potential^23^ (Fig 1J). This cohort included 8 tissue sections obtained from tumor tissues from 3 patients with confirmed diagnosis of GBM (described in methods). Microglia are one of the most strongly associated non-malignant cell types in regions with high expression of DNA repair pathway activities (Fig S1S–W). Neighborhoods rich in microglia are associated with higher DNA repair pathway expression. (Fig 1 K, L), suggesting that neoplastic cells in these regions might have an enhanced capacity to repair DNA damage following RT

### TAM-rich neighborhoods promote neoplastic cell DNA repair in patient brain tumors.

**Fig2.**
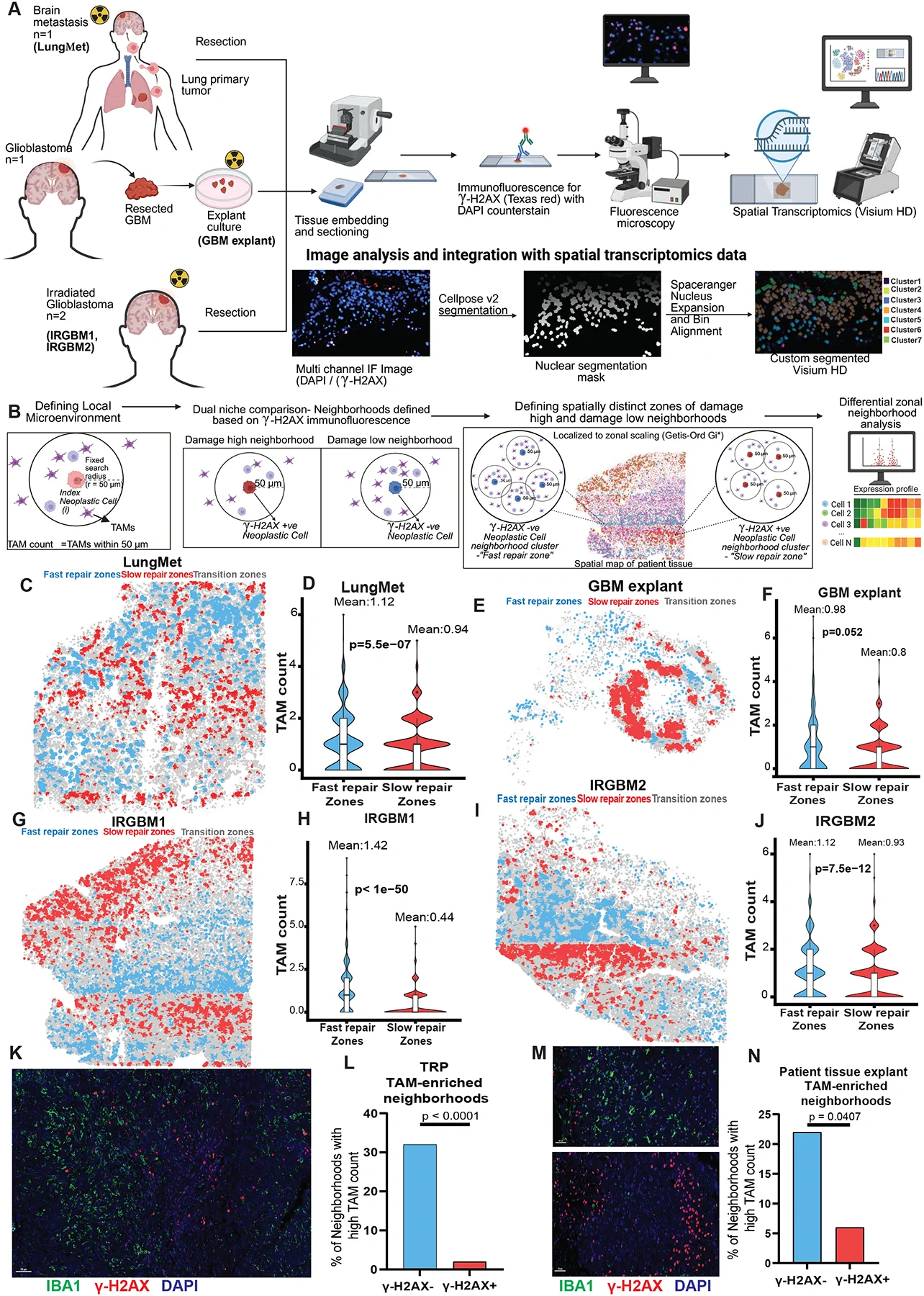
Rapid DNA repair in irradiated human brain tumors corresponds to spatial distribution of tumor associated macrophages. **A.** Illustration depicting the sample collection and processing workflow for HD spatial transcriptomics of human tissues. **B.** Illustration showing definition of neighborhoods and custom DNA repair zones analyzed in this study. **C-J.** Spatial map of lung metastasis to the brain(C), GBM explant(E), IRGBM1 (G), IRGBM2 (I) showing spatially distinct fast repair and slow repair zones. Graphical representation of Tumor associated macrophage (TAM) counts in fast repair and slow repair zones in LungMet tissue (D), GBM explant tissue (F), IRGBM1 (H), IRGBM2 (J). P-value calculated using Wilcoxon test. **K.** Representative image of multiplex immunofluorescence showing spatially distinct IBA1^+^ TAMs and γ-H2AX^+^ cells in TRP tissue 6hrs after RT (4Gy, scale bar 50um). **L.** Quantification of proportion of TAM enriched γ-H2AX positive vs. negative neighborhoods in TRP tissue 6hrs after radiation. P-value calculated using Fisher’s exact test. **M.** Representative images of multiplex immunofluorescence showing spatially distinct IBA1^+^ TAMs (top) and γ-H2AX^+^ cells (bottom) in GBM explant tissue 6hrs after radiation exposure (scale bar 50um). **N.** Quantification of proportion of TAM enriched γ-H2AX positive vs. negative neighborhoods in GBM explant tissue 6hrs after RT (12 Gy, scale bar 50μm). P-value calculated using Fisher’s exact test.

We next sought to understand the spatial regulation of RT-induced DNA damage in several types of patient brain tumors including GBM. Because GBM tumors are not typically irradiated prior to resection, we first utilized a short-term explant culture (**GBM explant**, Fig 2A) from a patient undergoing resection for an untreated GBM, which we irradiated (12 Gy in a single fraction) and then fixed and analyzed 6 h later.

Unlike patients with GBM, patients with brain *metastases* from extracranial cancers are sometimes treated with RT immediately prior to brain tumor resection to prevent local recurrence and leptomeningeal relapse^24,25^. Because brain metastases also harbor a complex non-neoplastic cell microenvironment including macrophages^26^, we used this preoperative RT treatment paradigm as an opportunity to study DNA repair in human patients. We identified a patient with metastatic lung cancer who developed a brain metastasis and was treated with preoperative RT (18 Gy in a single fraction) followed by resection of the metastasis the following day (**LungMet)**.

The emerging treatment paradigm of pre-operative RT for brain metastases also provided an unexpected opportunity to study GBM DNA repair in patients. Up to 10% of patients undergoing resection for a presumed brain metastasis (usually diagnosed on MRI imaging alone) are later found to have a different diagnosis based on pathologic analysis of the resected tumor tissue^27^. We thus identified two patients thought to have brain metastases (based on MRI findings and a history of extracranial cancer) who underwent pre-operative radiation followed by surgical resection whose tumors were ultimately diagnosed as GBM. While the treatment regimens varied (9 Gy × 3 daily fractions of RT followed by resection one day later **for IR GBM1**, 18 Gy in a single fraction of RT followed by resection three days later for **IR GBM2**), these rare tissues provided an opportunity to study acute GBM RT responses and DNA repair in the natural human brain environment.

We then analyzed these four irradiated tissues with combined γ-H2AX immunofluorescence and spatial transcriptomics as shown in Fig 2A. Advances in spatial transcriptomic technology coupled with our custom nuclear segmentation method allowed us to analyze these precious tissues with near cellular resolution, as opposed to the neighborhoods used in Fig 1 (Visium HD CytAssist CG000685). We identified neoplastic cells and the different non-neoplastic cell types in these tissues based on their transcriptional profiles, using a large human glioblastoma and adjacent cortex dataset^28^ as a reference and additionally confirmed the neoplastic cell annotation in the LungMet tissue using genes commonly overexpressed in lung cancer including EIF3^29–31^, KRT7^32^, SFTPB^33,34^, S100A14^35,36^. We then annotated the neoplastic cell population as positive or negative for γ-H2AX based on immunofluorescence (Fig 2A–B). The higher resolution of this spatial transcriptomic technique allowed us to first investigate neoplastic cell-intrinsic regulators of γ-H2AX resolution after RT. For the three GBM tissues, we assessed neoplastic cell states according to Neftel et. al^37^. and observed no differences in γ-H2AX staining between states (Figs S2A–C). Because stem-like neoplastic cells are postulated to have enhanced DNA repair and RT resistance^38,39^, we assessed transcriptional markers of stemness in γ-H2AX high and low neoplastic cell populations and found variable proportions in the 4 tissues we analyzed (Fig S2D). The association of stemness and DNA repair was variable across tissues with the proportion of stem-like cells higher in the γ-H2AX low (fast repair) neoplastic cells in IRGBM1 and IRGBM2, while the opposite was observed in LungMet and GBM explant.

Since spatial heterogeneity in DNA repair was seen in all the tissues studied, we then turned our study to neoplastic cell-extrinsic regulators of γ-H2AX positivity as in Fig 1, to identify potential common factors across all 4 tissues. To identify non-neoplastic cell neighbors for the neoplastic cells, we first defined a neighborhood radius by assessing the number of cells present within different radii ranging from 25um to 250um around each neoplastic cell and the extent of overlap between neighborhoods for each radius. A radius of 50 μm contained an average of around 3 cells with the least amount of overlap between neighborhoods. However, despite the small radius, over 90% of the neighborhoods overlapped, frequently leading to the same non-neoplastic cell being assigned to multiple neighborhoods with differing γ-H2AX positivity in the index neoplastic cells. To overcome this, we defined non-overlapping “zones” of rapid DNA repair (clusters of γ-H2AX negative neighborhoods) or slow DNA repair (clusters of γ-H2AX negative neighborhoods) using local Getis-Ord G_î* statistic^40^ which identified continuous stretches of overlapping γ-H2AX positive or negative neighborhoods (Fig 2B). The transition zones containing overlapping γ-H2AX positive and negative neighborhoods were not included in downstream analysis.

In all four tissues, we observed spatially distinct γ-H2AX positive and negative zones throughout the irradiated tissue (Fig 2C, 2E, 2G and 2I). To assess how non-malignant cells might regulate neoplastic cell γ-H2AX resolution, we compared the non-neoplastic cell composition within the fast and slow repair zones. From this analysis, we confirmed that TAMs are the most abundant non-malignant cell population across all four irradiated tissues (Fig S2E) and show that TAM abundance was elevated in fast repair zones (Fig 2D,F,H,J). Other cell types, including astrocytes, neurons and oligodendrocytes showed inconsistent trends, whereas oligodendrocyte precursor cells (OPCs) were enriched in zones with slow repair (Fig S2F–I). These findings suggest that proximity to TAMs is associated with rapid repair of RT-induced DNA damage in human brain tumors.

We then sought to confirm these findings with an orthogonal technique. We co-stained irradiated intracranial murine GBM tissue (TRP, as in Fig 1) and irradiated human GBM explant tissue (as in Fig 2E) for γ-H2AX and the TAM marker IBA1 using multiplex IF (fig 2K,M). We defined non-overlapping circular regions of interest (ROIs) containing approximately 50 cells each. We assessed γ-H2AX positivity in IBA1^−^ (i.e., non-TAM) cells. ROIs in which fewer than 10% of the non-TAM cells were γ-H2AX^+^ were defined as γ-H2AX negative and ROIs in which ≥10% of non-TAM cells were γ-H2AX^+^ were defined as γ-H2AX positive neighborhoods. In both irradiated intracranial murine GBM tumors (Figs 2K–L) and human GBM explants (Figs 2M–N), there was elevated TAM content in γ-H2AX negative neighborhoods. Together, these immunofluorescence and spatial transcriptomic analyses suggest that proximity to TAMs promotes neoplastic cell DNA repair in GBM.

### Microglia promote the resolution of RT-induced DNA damage marks in GBM cells.

**Fig3.**
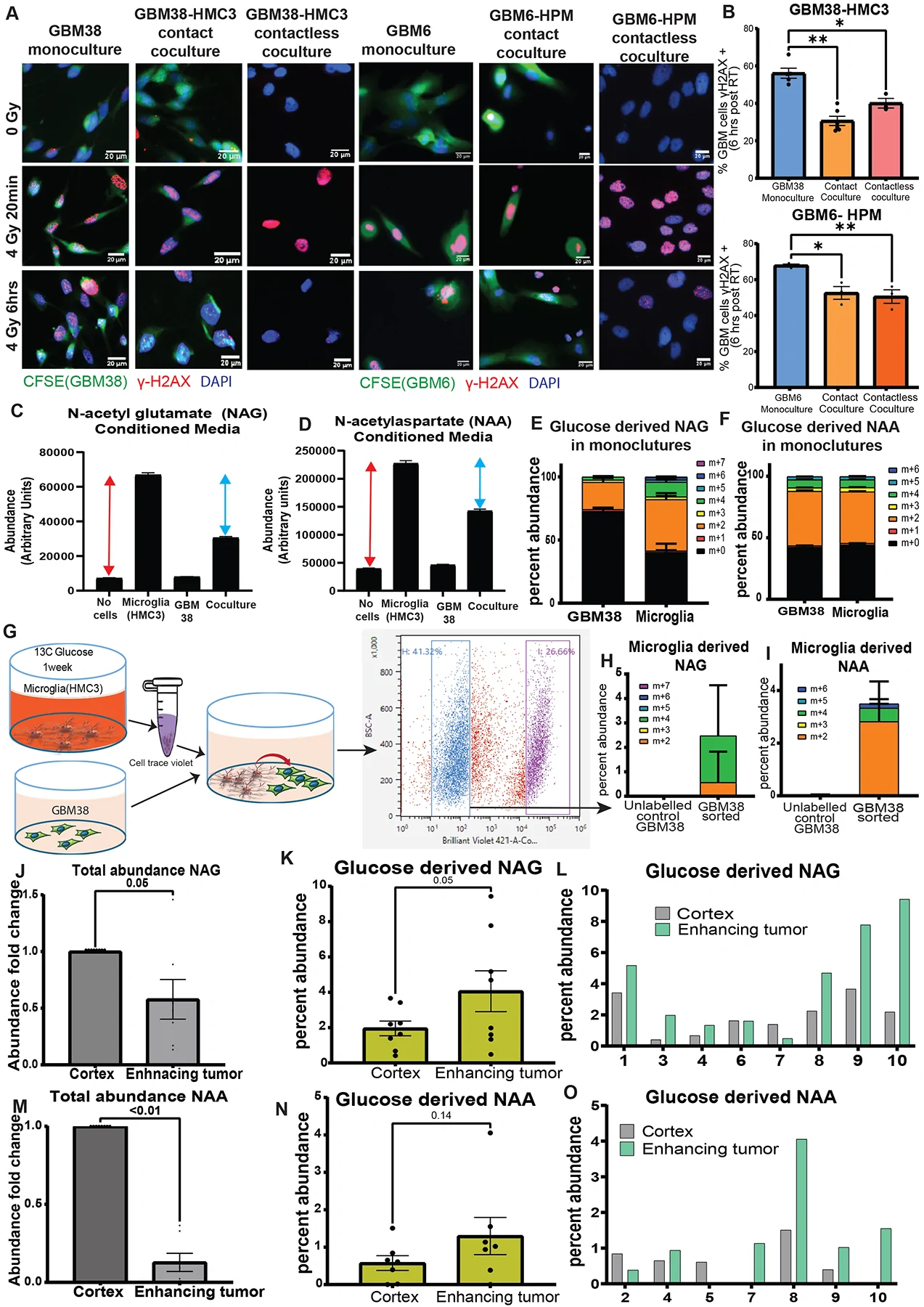
Microglia secrete N-acetyl glutamate and N-acetyl aspartate, which are consumed by glioblastoma cells. **A.** Representative images of γ-H2AX immunofluorescence (red) in monocultures and cocultures of GBM38 and HMC3 cells and GBM6 and HPM cells (as indicated). CFSE (green fluorescence) marks GBM cells in monococulture and contact coculture of GBM cells and microglia. In contactless coculture, cells shown in the image are GBM cells from the contactless coculture system. **B.** Quantification of GBM cells that remain γ-H2AX^+^ 6 hrs after exposure to radiation (4 Gy) in the monoculture and coculture systems, as indicated. P values calculated using one-way Anova (n=5 [obtained from 2 experiments] for GBM38 monoculture, n=6 [2 experiments, 3 replicates each] for GBM38-HMC3 contact cocultures and n=3 for GBM38-HMC3 contactless cocultures; n=3 for each condition in GBM6-HPM coculture experiment). **C.** Abundance of N-acetyl glutamate (NAG) in conditioned media of monocultures and coculture of GBM38 and HMC3 microglia cells showing secretion by microglia (red arrow) and reduction in abundance in cocultures (blue arrow). (n=5 biologic replicates for each condition from a single experiment.) **D.** Abundance of N-acetyl aspartate (NAA) in conditioned media of monocultures and coculture of GBM38 and HMC3 microglia cells showing secretion by microglia (red arrow) and lower abundance in cocultures (blue arrow). (n=5 biologic replicates for each condition. **E.** Percent abundance and labelling pattern of intracellular ^13^C_6_ glucose-derived NAG in GBM38 and HMC3 monocultures (n=3 biologic replicates). F. Percent abundance and labelling pattern of intracellular 13C6 glucose derived NAA in GBM38 and HMC3 monocultures (n=3 biologic replicates). G. Illustration depicting the experimental design where HMC3 cells were first cultured in 13C6 glucose media for 1 week, then fluorescently labeled with celltrace violet, followed by coculture with GBM38 cells in media without 13C tracer, followed by flow cytometry-based isolation of GBM38 cells (celltrace violet negative) from coculture. H. Percent abundance of microglia derived 13C labelled NAG in GBM38 cells.(n=3 unlabeled controls, n=5 replicates of GBM cells sorted from coculture with microglia precultured with 13C6 glucose.) I. Percent abundance of microglia derived 13C labelled NAA in GBM38 cells. (n=3 unlabeled controls, n=5 replicates of GBM cells sorted from coculture with microglia precultured with 13C6 glucose.) J. Total abundance fold change of NAG in human glioblastoma tumor tissues, each compared to its adjacent cortex (n=8). P values calculated using paired T-test. K, L. Percent abundance of 13C6 glucose-derived NAG in human GBM tissues compared to adjacent cortex. P values calculated using paired T test. M. Total abundance fold-change of NAA in human glioblastoma tumor tissues compared to their adjacent cortices (n=8). P values calculated using paired T test. N, O. Percent abundance of 13C6 glucose-derived NAA in human GBM tissues compared to adjacent cortices. P values calculated using paired T test. P values indicated as * 0.01 to 0.05, ** 0.001 to 0.01, *** 0.0001 to 0.001, ****<0.0001. All error bars show standard error of mean (SEM).

The above studies suggested that proximity to TAMs is associated with the repair of RT-induced DNA damage in GBM tumors. To assess the causality of this association, we turned to reductionist laboratory models of GBM and microglia, which are the brain specific TAMs. We cocultured human GBM cells (GBM38 or GBM6) *in vitro* with human microglia cells (HMC3 or HPM cells) either in contact with each other or in a trans-well culture without contact between cell types (Fig S3A). We irradiated monocultures and cocultures and assessed DNA repair after RT using γ-H2AX immunofluorescence (IF). As in *in vivo* GBMs (Fig 1B), most neoplastic cells stained positive for γ-H2AX shortly after RT whether cultured in isolation, in contact with microglia, or with contactless microglia coculture (Fig 3A, 20 min timepoint, Fig S3B). By contrast, the fraction of GBM cells that remain positive for γ-H2AX 6hrs after RT is significantly lower when co-cultured with microglia (with or without contact, Fig 3A,B). These findings suggest that microglia may promote GBM cell DNA repair through a secreted factor.

### N-acetylated amino acids are secreted by microglia and consumed by GBM cells.

To identify potential secreted factors mediating microglia-induced DNA repair in GBM cells, we reanalyzed high-definition spatial transcriptomic data (Fig 2) to identify all ABC and SLC family transporters upregulated in the TAMs present in the γ-H2AX negative zones of irradiated human GBMs. This analysis showed an upregulation of amino acid and carboxylic acid transporters in the TAMs mediating rapid neoplastic cell DNA repair (Fig S3C), which suggested metabolic exchange between TAMs and neoplastic glioma cells.

We then performed metabolomic analysis on conditioned media from isolated glioma cells, isolated microglia, and co-cultures, to identify microglia-derived metabolites which could be utilized by neoplastic cells to facilitate repair of RT-induced DNA damage (Fig S3D). Some metabolites (such as glutamine) were consumed from the media in all culture conditions (glioma monoculture, microglia monoculture, microglia/glioma co-culture, while others (such as ketovaleric acid) were secreted in all conditions (Fig S3E). However, we were interested in identifying metabolites that were elevated in the conditioned media of isolated microglia (red arrow, Fig 3C,D), not elevated in the conditioned media of isolated glioma cells, and minimally elevated in the conditioned media from glioma/microglia co-cultures (blue arrow, Fig 3C,D), as we hypothesized this pattern would represent secretion of a metabolite from microglia and uptake by neoplastic cells. Searching with this pattern in mind, we identified N-acetyl Glutamate (NAG) and N-acetyl Aspartate (NAA) as the top candidate metabolites that appeared to be secreted by microglia and utilized by glioblastoma cells (Fig 3C,D, Fig S3E). We obtained consistent results in a similar co-culture experiment with different models of microglia and patient-derived GBM (Fig S3F,G).

NAG and NAA are neuroactive metabolites with diverse physiologic functions^41,42^. Structurally, NAG and NAA are simply the amino acids glutamate and aspartate modified by a 2-carbon acetyl group at their N-termini, which is typically donated by acetyl-coA. Since the immediate precursors of both NAG and NAA can be derived from glucose, we interrogated their synthesis in microglia and GBM cells using stable isotope tracing with ^13^C_6_-Glucose. The pathways through which glucose carbons can be utilized to synthesize NAG and NAA are depicted in Fig S3H. Both cell types can synthesize NAG and NAA from glucose to varying degrees (Fig 3E,F). We then designed an experiment to directly assess whether NAG and NAA synthesized by microglia could be transferred to GBM cells in coculture (Fig 3G). We cultured microglia in ^13^C_6_-Glucose media for 1 week to maximize ^13^C labeling of intracellular metabolites including NAG/NAA, washed away ^13^C_6_ glucose tracer, and fluorescently labelled them using cell trace violet. These fluorescent, ^13^C-labeled, microglia were then co-cultured with non-fluorescent, non-^13^C-labeled GBM cells in unlabeled complete media. GBM cells were isolated using flow cytometry and analyzed by mass spectrometry, which revealed ^13^C_6_-Glucose derived NAG and NAA in GBM cells (Fig 3H,I). We did not detect ^13^C labeling of unacetylated aspartate or glutamate in these GBM cells(Fig S3 I,J), suggesting that the detected ^13^C NAG and ^13^C NAA are not a result of synthesis in the GBM cells from potential transferred unacetylated glutamate or aspartate. These results suggest that microglia secrete NAG and NAA, which are then consumed by GBM cells.

### NAG and NAA synthesis are active in patient GBMs.

Having established that NAG and NAA are synthesized from glucose by GBM cells and microglia in vitro, we assessed their synthesis *in vivo* in human GBM tissues by analyzing data from our human ^13^C_6_-Glucose stable isotope tracing cohort^5^. Patients undergoing surgical resection for brain tumors received ^13^C_6_-Glucose infusion. Tissue obtained from the surgery was analyzed for glucose-derived metabolite abundance and labeling patterns. Only the tumor tissues with confirmed histomolecular diagnosis of GBM were included in this study. Five of these samples were from our previously published study^5^ while three are newly analyzed. NAG was detected in 8 samples while NAA was detected in 7 samples. Analysis of ^13^C-labeling of NAG and NAA in these samples revealed that despite having a lower total abundance of NAG and NAA in GBM tumor compared to the surrounding cortex (Fig 3J,M), there is elevated abundance of ^13^C-labeled NAG in tumor compared cortex (Fig 3K) and a non-significant trend (p=0.1) towards elevation of ^13^C-labeled NAA in tumor (Fig 3N). On a per-patient basis, 6 of 8 GBM patients had elevated ^13^C-labeled NAG in tumor compared to cortex (Fig 3L), while 5 of 7 GBM patients had elevated ^13^C-labeled NAA in tumor compared to cortex (Fig 3O). These findings indicate active NAG and NAA synthesis from glucose in cellularly heterogeneous human GBM tumors.

### NAG and NAA supplementation independently induce rapid DNA damage repair and radioresistant phenotypes in GBM cells.

We next sought to understand whether secreted NAG and/or NAA could be related to the rapid DNA repair conferred to neoplastic cells by microglia. Treatment with RT increased levels of NAG and NAA in glioma cells (Fig S3K,L), suggestive of a role in the RT response. To test whether NAG and NAA independently promote rapid RT-induced DNA damage repair in GBM, we performed post RT γ-H2AX immunofluorescence with supplemented NAG or NAA. We evaluated a range of serially diluted NAG concentrations in two patient-derived GBM cell lines, GBM6 and GBM38. While NAG at 1 μM had minimal effects, NAG at concentrations of 10–100 μM (which is similar to the 30–40 μM range measured in rodent brains^42^) promoted the resolution of RT-induced γ-H2AX immunofluorescence (Fig S4A,B), thus we studied 100μM as our typical NAG concentration moving forward. For NAA supplementation, 1mM concentration of NAA was used for all experiments based on reported physiological concentration in the human brain^41,43^.

**Fig4.**
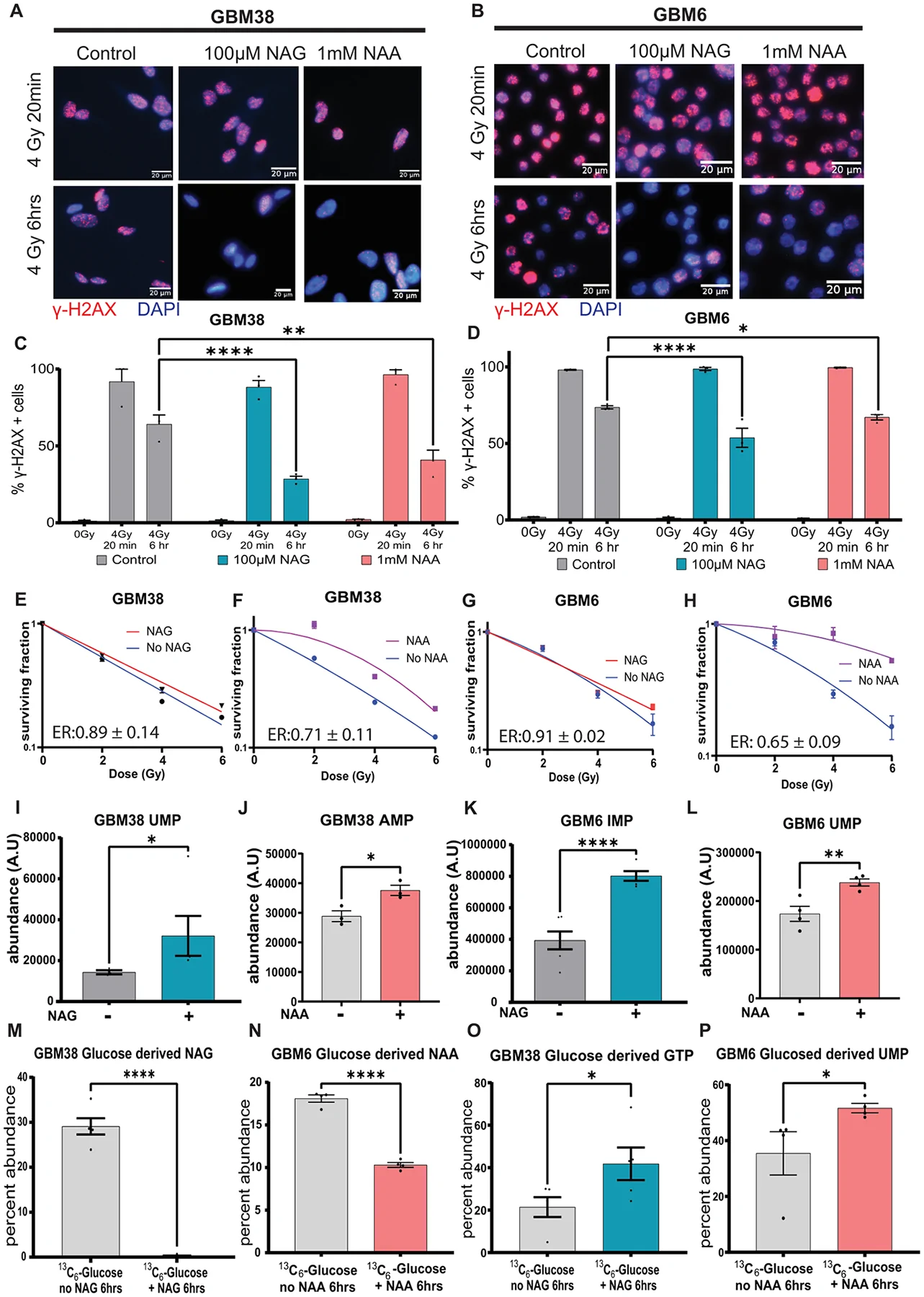
N-acetyl glutamate and N-acetyl aspartate enhance DNA repair and nucleotide synthesis in glioblastoma cells. **A-B.** Representative images of γ-H2AX immunofluorescence in GBM38 (A) or GBM6 (B) cells supplemented with NAG or NAA 20 min and 6hrs after RT (4 Gy) exposure. **C-D.** Quantification of γ-H2AX positivity of GBM38 (C) or GBM6 (D) cells supplemented with NAG or NAA in unirradiated cells and irradiated cells 20 min and 6hrs after RT (4 Gy). **E-F.** Clonogenic survival assay showing surviving fraction of GBM38 cells supplemented with NAG (E) or NAA (F). ER indicates the enhancement ratio compared to control cells with error bars indicating SEM from 3 biologic replicates in one experiment. ER was calculated from 3 separate experiments, each with 3 biologic replicatesand ER ± SEM is shown in the figure. ER<1 indicates radioprotection **G-H.** Clonogenic survival assay showing surviving fraction of GBM6 cells supplemented with NAG (G) or NAA (H). ER indicates the enhancement ratio compared to control cells from 3 biologic replicates in one experiment. ER was calculated from 3 separate experiments, each with 3 biologic replicates and mean ER ± SEM is shown in the figure. ER<1 indicates radioprotection **I-L.** Comparison of intracellular abundance of nucleotides in GBM38 and GBM6 cells supplemented with NAG or NAA as indicated(n=3 biologic replicates in GBM38 no NAG control and n=5 in GBM38 NAG supplemented condition(I), n=3 biologic replicates for each condition in GBM38±NAA experiment(J), n= 6 biologic replicates for GBM6 no NAG control, n=5 for GBM6 NAG supplemented condition(K), n=4 biologic replicates for each condition in GBM6 ± NAA experiment (L). M. Comparison of percent abundance of glucose derived-NAG in GBM38 cells supplemented with exogenous unlabeled NAG (n=5 biologic replicates for each condition). N. Comparison of percent abundance of 13C6 glucose derived-NAA in GBM6 cells supplemented with exogenous unlabelled NAA(n=4 biologic replicates for each condition). O, P. 13C6 glucose derived nucleotide percent abundance in GBM38 and GBM6 cells supplemented with NAG or NAA as indicated(n=5 biologic replicates for each condition in O and n=4 biologic replicates for each condition in P). P values indicated as * 0.01 to 0.05, ** 0.001 to 0.01, *** 0.0001 to 0.001, ****<0.0001. Error bars depict SEM in all graphs.

Using two different patient-derived GBM cell line models (GBM38, Fig 4 A,C; GBM6, Fig 4 B,D), we observed that neither NAG nor NAA supplementation affected the induction of γ-H2AX staining in cells analyzed minutes after RT. However, NAG supplementation alone, as well as NAA supplementation alone enhanced the resolution of glioma cell γ-H2AX staining 6 hr after RT. These findings mirror those seen with microglia co-culture (Fig 3A,B) and suggest that environmental NAG and NAA may independently promote repair of RT-induced DNA damage in GBM cells.

Enhanced repair of RT-induced DNA damage promotes cancer treatment resistance by conferring increased survival following RT. To further evaluate the functional consequence of NAG or NAA supplementation on radiation responses, we performed clonogenic survival assays with and without NAG/NAA supplementation. In agreement with the results of the IF experiment, NAA supplementation significantly increased GBM colony survival after irradiation while NAG supplementation moderately increased GBM colony survival after irradiation (Fig. 4E–H). These results show that NAG and NAA can each enhance DNA damage repair and promote radiation resistance in GBM cell lines.

### N-acetylated amino acids boost nucleotide synthesis in GBM cells

To begin to understand the metabolic mechanisms underlying NAG and NAA-mediated enhanced DNA repair, we examined intracellular metabolic alterations in GBM cells following exogenous NAG or NAA supplementation. We performed metabolomic profiling of GBM cells 6 hours after treatment with 100 μM NAG or 1mM NAA, compared to untreated controls. We first confirmed that intracellular NAG and NAA levels were elevated following exogenous supplementation of NAG and NAA, respectively, suggesting active uptake and retention of NAG and NAA by GBM cells (Fig S4 C,D,G,H blue box in E, red box in F). Both NAG supplementation and NAA supplementation led to a marked increase in multiple nucleotide intermediates, including adenosine 5′-monophosphate (AMP), uridine 5′-monophosphate (UMP), inosine 5′-monophosphate (IMP), and cytidine 5′-monophosphate (CMP), among others (Fig. 4I–L, Fig S4E,F). These findings indicate that exogenous NAG and NAA supplementation increases purine and pyrimidine pools in GBM cells.

We then performed tracing experiments to determine how NAG and NAA affected glioma cell metabolic activity. We first investigated the canonical function of NAG in cells, i.e., allosteric activation of the enzyme Carbamoyl Phosphate Synthetase 1 (CPS1), which is the rate-limiting step of Urea cycle^44–46^. We performed ^15^N Ammonium tracing in GBM38 and GBM6 cells(Fig S4I). We observed little to no urea cycle intermediate labelling in these cells and saw no difference between NAG supplemented cells and untreated cells (Fig S4J,K). However, we did observe the incorporation of ^15^N ammonium into glutamate (Fig S4L,M) and NAG (Fig S4 N–Q) in GBM cells, suggesting active synthesis of NAG from glutamate in GBM. This ammonium labeling of NAG drops (Fig S4 N–Q) when exogenous NAG is present, suggesting suppression of NAG synthesis when extracellular NAG sources are present.

To determine whether exogenous NAG can be further metabolized within GBM cells, we performed stable isotope tracing with ^13^C_5_-NAG. We saw uptake of NAG (Fig S4R) in GBM cells, but no conversion into glutamate (Fig S4S), thereby suggesting that there is no or minimal conversion of NAG into its breakdown products, acetate and glutamate(Fig S4 T). This was further supported by lack of increase in total glutamate abundance in the cells supplemented with NAG (Fig S4 U).

We then studied the canonical function of NAA, as seen in oligodendrocytes, i.e., conversion to acetate and aspartate to facilitate myelin synthesis^47–49^. Since aspartate can be used for nucleotide synthesis, we tested whether GBM cells also convert NAA to acetate and aspartate. We performed stable isotope tracing with ^13^C_4_ NAA in GBM cells and confirmed the uptake of NAA (Fig S4 V). Unlike NAG, we *did* observe conversion of NAA into non-acetylated aspartate (Fig S4 W) and downstream pyrimidines (FigS4 X, Y). The pathways involved in this process are illustrated in Fig S4 Z. However, we did not observe a significant increase in the abundance of aspartate (Fig S4AA).

Since our findings from ^15^N Ammonium tracing were suggestive of decreased NAG synthesis when exogenous NAG is supplemented, we hypothesized that the glucose used to endogenously synthesize NAG and NAA might be spared and rerouted for nucleotide synthesis when exogenous NAG or NAA are supplemented.

Supporting this hypothesis, ^13^C_6_-glucose tracing in GBM38 and GBM6 cells with or without exogenous unlabeled NAG or NAA supplementation revealed that glucose derived NAG and NAA significantly decreased in cells supplemented with exogenous NAG and NAA, respectively (Fig. 4M,N, Fig S4 AB,AC). NAG and NAA supplementation each significantly increased the abundance of glucose-derived nucleotides in GBM cells (Fig. 4O,P).

### NAG and NAA supplementation increase GBM acetyl CoA levels and acetylation of H2AX in GBM cells.

**Fig5.**
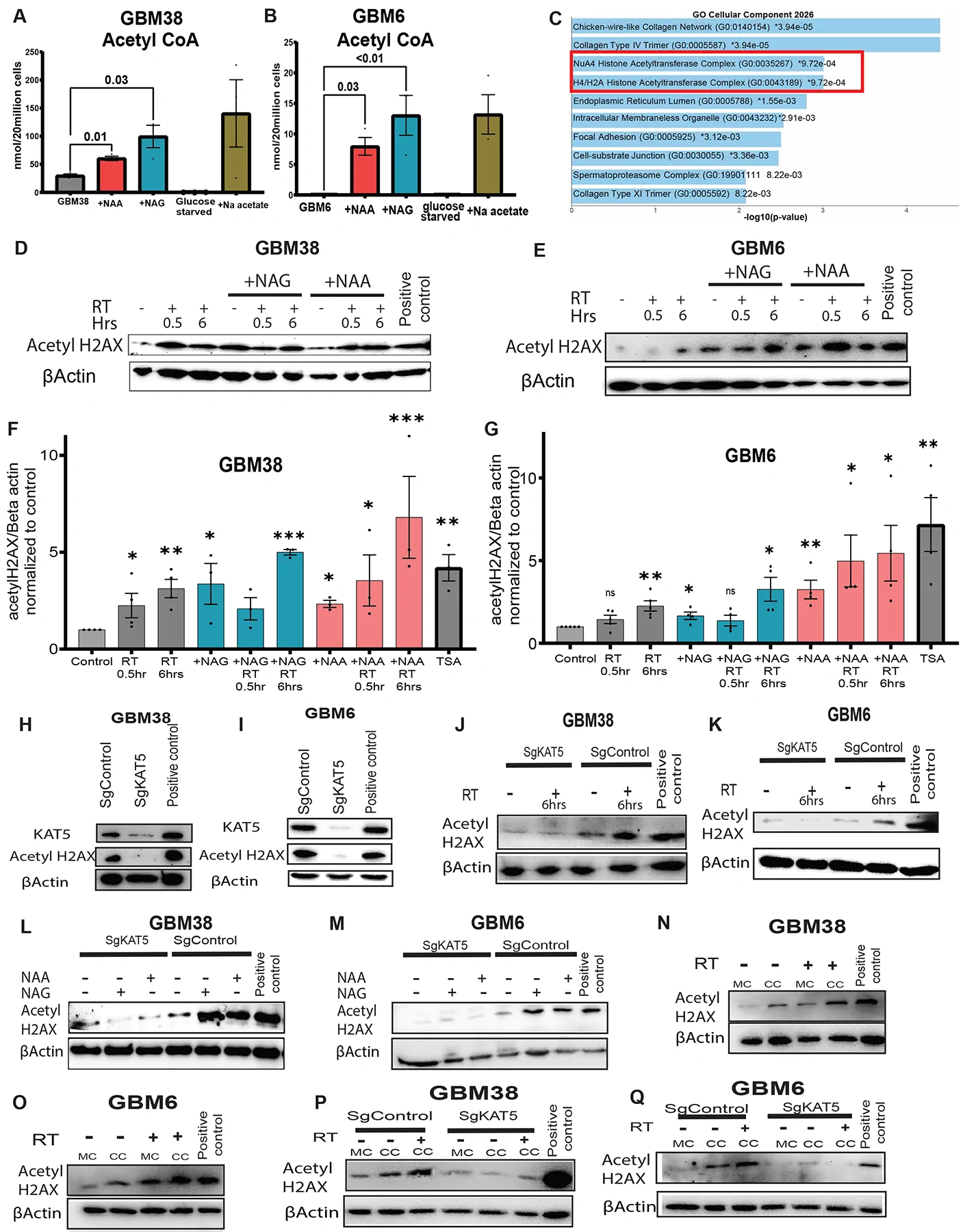
N-acetyl glutamate and N-acetyl aspartate enhance acetylation of H2AX via KAT5. **A.** Acetyl-CoA abundance in GBM38 cells supplemented with 100μM NAG or 1mM NAA or 4M Sodium acetate (positive control) or 12 hrs glucose starvation (negative control),(n=3 separate experiments each with 5 technical replicates and 3 biologic replicates). **B.** Acetyl-coA abundance in GBM6 cells supplemented with 100μM NAG or 1mM NAA or 4M Sodium acetate (positive control) or glucose starvation (negative control) (n=3 biologic replicates, each with 5 technical replicates for each condition). **C.** Gene ontology of commonly upregulated genes in neoplastic cells negative for γ-H2AX (damage low) in IRGBM1 and IRGBM2 tissues (compared to damage high neoplastic cells positive for γ-H2AX) from irradiated human brain tumor cohort described in Fig 2. **D,E.** Representative western blot showing higher acetylation of H2AX in GBM38 cells (D) and GBM6 cells (E) treated with NAG or NAA and/or RT (4 Gy). 0.3 μM Trichostatin A (TSA) treated cells served as positive control. **F,G.** Quantification of D and E in 4 replicates. P values calculated using one sample T-test. All conditions were compared to the control condition. **H,I.** Representative western blot from 4 separate experiments assessing acetylation of H2AX and KAT5 expression in control (sgControl) or KAT5 knockdown (sgKAT5) GBM38 cells(H) and GBM6 cells (I). Treatment of wild type GBM38 (in H) and GBM6 (in I) cells with TSA (0.3 μM for 24 hrs) was used as a + control. **J,K.** Representative western blot from 3 separate experiments assessing acetylation of H2AX of sgControl or sgKAT5 GBM38(J) and GBM6 (K) cells at baseline and 6hrs after RT (4 Gy). TSA treated (0.3 μM for 24hrs) sgControl cells were used as a positive control. **L,M. Representative** western blot assessing acetylation of H2AX of sgControl or sgKAT5 GBM38(L) and GBM6 (M) cells at baseline and after NAG (100μM) or NAA (1mM) supplementation (48hrs). TSA treated (0.3 μM for 24hrs) sgControl cells were used as a positive control. **N,O.** Representative western blot assessing acetylation of H2AX in wildtype GBM38 (N) and GBM6 (O) cells grown as monocultures(mc) or in contactless coculture (cc) with HMC3 microglia at baseline and 6hrs after RT (4 Gy). Treatment of wild type GBM38 cells (in N) and GBM6 cells (in O) with TSA (0.3 μM for 24hrs) was used as a positive control. **P,Q.** Representative western blot assessing acetylation of H2AX of sgControl or sgKAT5 GBM38 cells (P) and GBM6 cells (Q) grown as monocultures(mc) or in contactless coculture (cc) with HMC3 microglia at baseline and 6hrs after RT (4 Gy). TSA treated (0.3 μM for 24hrs) sgControl cells were used as a positive control. P values indicated as * 0.01 to 0.05, ** 0.001 to 0.01, *** 0.0001 to 0.001, ****<0.0001

While increased nucleotides can enhance GBM DNA repair^3,4,50–52^, the striking glucose sparing affect of NAG and NAA supplementation prompted us to explore other mechanisms. Acetyl-CoA, a critical molecule for DNA repair and GBM biology, is used to synthesize both NAG and NAA^53–57^. We hypothesized that a ready source of environmental NAG and NAA could decrease Acetyl-CoA consumption and thus promote DNA repair. Similarly, the ability of glioma cells to split the NAA molecule into its aspartate and acetate components (Fig S4 W,Z), could elevate acetyl CoA pools. We therefore quantified acetyl CoA levels in GBM cells following NAG or NAA supplementation using glucose starvation as a negative control and acetate supplementation as a positive control^58^. Both NAA and NAG supplementation elevated acetyl CoA levels in multiple GBM cell lines (Fig 5A,B).

Acetyl CoA is utilized for numerous steps in the DNA damage response and is a potential link between NAG/NAA and DNA repair. Revisiting our human tumor spatial transcriptomics data (Fig 2G,I) revealed that the GBM cells that quickly become γ-H2AX low after RT (fast repair) commonly upregulated histone acetyltransferase gene expression (Fig 5C). Several studies have shown that increased availability of acetyl-coA boosts histone acetylation^58–63^ and that KAT5(also known as Tip60) mediated acetylation of H2AX is essential for DNA repair activity^64–68^. These findings prompted us to ask whether supplementation of NAG or NAA leads to increased acetylation of H2AX, a post-translational mark needed for facilitating DNA repair after RT^64^. Supplementation of GBM cells with NAG or NAA increased baseline H2AX acetylation in both GBM38 and GBM6 cell lines (Fig 5D–G). Both cell lines increased the acetylation of H2AX following RT, and this RT-induced acetylation of H2AX was enhanced in both cell lines when environmental NAG and NAA were present (Fig 5D–G). Wildtype cells treated with 0.3 μM Trichostatin A (TSA), a potent histone deacetylase inhibitor were used as the positive controls for these experiments. These findings suggest that the presence of exogenous NAG and NAA can enhance the availability of intracellular acetyl CoA and facilitate H2AX acetylation.

### KAT5 knockdown reverses NAG and NAA mediated fast DNA repair and radiation resistance phenotypes in GBM cells.

We next sought to interrupt this biology to determine whether NAG and NAA exert their protective effects on glioma directly via histone acetylation. KAT5 is the enzyme that catalyzes the transfer of acetate from acetyl-coA to H2AX^69^. To study whether knock down of KAT5 leads to reversal of NAG and NAA mediated acetylation of H2AX and subsequent DNA repair advantage, we generated CRISPR-CAS9 gene edited KAT5 knockdown clones of GBM38 and GBM6 cells. Knockdown of KAT5 reduced acetylH2AX in both GBM cell lines (Fig5 H,I). The RT-induced increase in acetyl H2AX in wild type GBM cells is absent in the KAT5 knockdown cells (Fig5J,K). Similarly, KAT5 knockdown reversed the ability of NAG and NAA to enhance H2AX acetylation (Fig 5L,M).

We then tested whether coculture with microglia replicates the effect of NAG and NAA on acetylation of H2AX. We cocultured microglia (HMC3 cells) with GBM38 or GBM6 wild type cells in a contactless coculture system and compared the acetylation of H2AX in GBM cells. There is increased acetylation of H2AX in GBM cells cocultured with microglia when compared to monocultures (Fig5 N,O). Post RT, the increase in acetylation is more pronounced in the GBM cells cocultured with microglia (Fig5 N,O). We then tested whether knockdown of KAT5 reverses this increase in acetylation by coculturing sgcontrol or sgKAT5 cells in both cell lines with microglia. sgKAT5 cells did not show any change in acetylation of H2AX when cocultured with microglia or after RT in coculture (Fig 5 P,Q). From this, we confirmed that KAT5 knockdown reverses microglia induced increase in acetylation of H2AX.

**Fig6.**
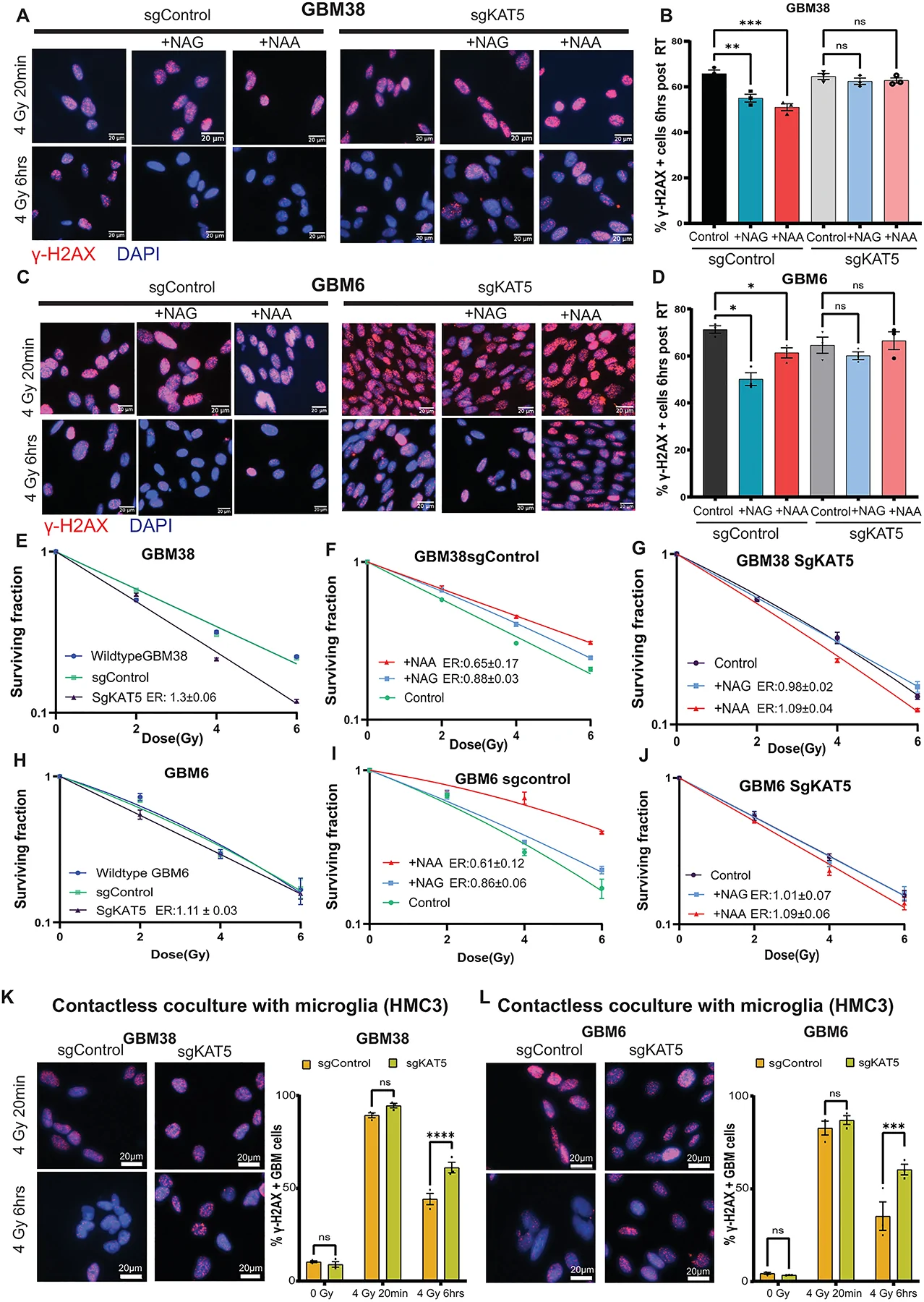
KAT5 knockdown reverses N-acetyl glutamate and N-acetyl aspartate - mediated DNA repair and radiation resistance. **A.** Representative images of γ-H2AX immunofluorescence in GBM38 CRISPR control (sgControl) and KAT5 Knockdown (sgKAT5) GBM38 cells supplemented with NAG or NAA 20 min and 6hrs after RT exposure (4 Gy). **B.** Quantification of γ-H2AX positivity in sgControl and sgKAT5 GBM38 cells supplemented with NAG or NAA 6hrs after RT (4 Gy). (n=3 separate experiments, each with 3 biologic replicates for each condition) P values calculated using unpaired T tests. Error bars indicate SEM **C.** Representative images of γ-H2AX immunofluorescence in GBM6 CRISPR control (sgControl) and KAT5 Knockdown (sgKAT5) GBM6 cells supplemented with NAG or NAA 20 min and 6hrs after RT exposure (4 Gy). **D.** Quantification of γ-H2AX positivity in sgControl and sgKAT5 GBM6 cells supplemented with NAG or NAA 6hrs after RT (4 Gy). (n=3 biologic replicates for each condition) P values calculated using unpaired T tests. Error bars indicate SEM. **E-J.** Clonogenic survival curves showing surviving fractions of cells following exposure to different doses of RT (0–6 Gy) in unmodified (wildtype), CRISPR control (sgControl) and KAT5 knockdown (sgKAT5) GBM38 (E-G) and GBM6 (H-J) cells. In F&G and I&J, cells were supplemented with 100 μM NAG or 1mM NAA and replenished every 48 hrs. Mean enhancement ratio (ER) calculated from 3 separate experiments with 3 biologic replicates each ± SEM is shown in the figures. Error bars show SEM. **K,L.** Representative images of γ-H2AX immunofluorescence in CRISPR control (sgControl) and KAT5 Knockdown (sgKAT5) GBM38 cells (K) and GBM6 cells (L) cocultured with HMC3 microglia for 48 hrs followed by RT (4 Gy) 20min and 6 hrs after exposure. Quantification of γ-H2AX positive cells in these GBM cells in unirradiated (0Gy), and irradiated (4 Gy) conditions, 20min and 6hrs after RT. (n=3 biologic replicates) P values calculated using unpaired T test. P values indicated as * 0.01 to 0.05, ** 0.001 to 0.01, *** 0.0001 to 0.001, ****<0.0001

To determine whether the interruption of H2AX acetylation reversed the DNA repair advantage conferred by NAG and NAA, we irradiated sgCon and sgKAT5 cells in the presence or absence of NAG/NAA and assessed DNA repair using immunofluorescence. Consistent with prior results (Fig 4A–D), supplementation of NAG/NAA to sgControl GBM38 and GBM6 cells promoted the resolution of γ-H2AX foci 6 hrs after RT without noticeably affecting the early induction of γH2AX after RT (Fig 6A–D, FigS5 A,B). However, these effects were lost in sgKAT5 GBM38 or GBM6 cells with reduced levels of KAT5 (Fig 6A–D). To rule out the possibility that NAG and NAA affected posttranslational modifications on H2AX without affecting downstream DNA repair, we also assessed 53BP1 foci as in Fig S1M,N. While NAG and NAA supplementation enhanced the resolution of 53BP foci in sgControl GBM38 cells (Fig S5C,E)), they had minimal effects in sgKAT5 GBM38 cells (Fig S5D,E).

Seeing that reducing expression of KAT5 reduced the ability of NAG and NAA to promote DNA repair in GBM cells, we next assessed how reduction of KAT5 affected survival after RT and/or NAG/NAA supplementation. KAT5 knockdown alone modestly radiosensitized GBM38 (Fig 6E) and GBM6 (Fig 6H) cells, as has been observed in other models^70^. As seen in our earlier studies, NAA and NAG both protected sgControl GBM6 and GBM38 cells from RT, with more pronounced effects seen with NAA (Fig 6F,I). However, the protective effects of both NAA and NAG were lost in GBM38 and GBM6 cells lacking KAT5 (Fig 6G,J).

We then tested whether coculture with microglia show reduced ability to promote DNA repair in the sgKAT5 cells, we cocultured microglia (HMC3 cells) with GBM38 or GBM6 sgControl cells and sgKAT5 cells in a contactless coculture system and compared the resolution of γH2AX 6hrs after RT in the GBM cells. While coculture with microglia promoted DNA repair in the sgControl cells as expected, there was significantly higher proportion of remaining DNA damage in the sgKAT5 cells cocultured with microglia(Fig 6K,L). Because sgKAT5 had minimal effects on γH2AX positivity after RT in glioma monocultures (Fig 6B,D), these results indicate that KAT5 knockdown in GBM cells reverses the fast DNA repair phenotype conferred by microglia.

**Figure 7.**
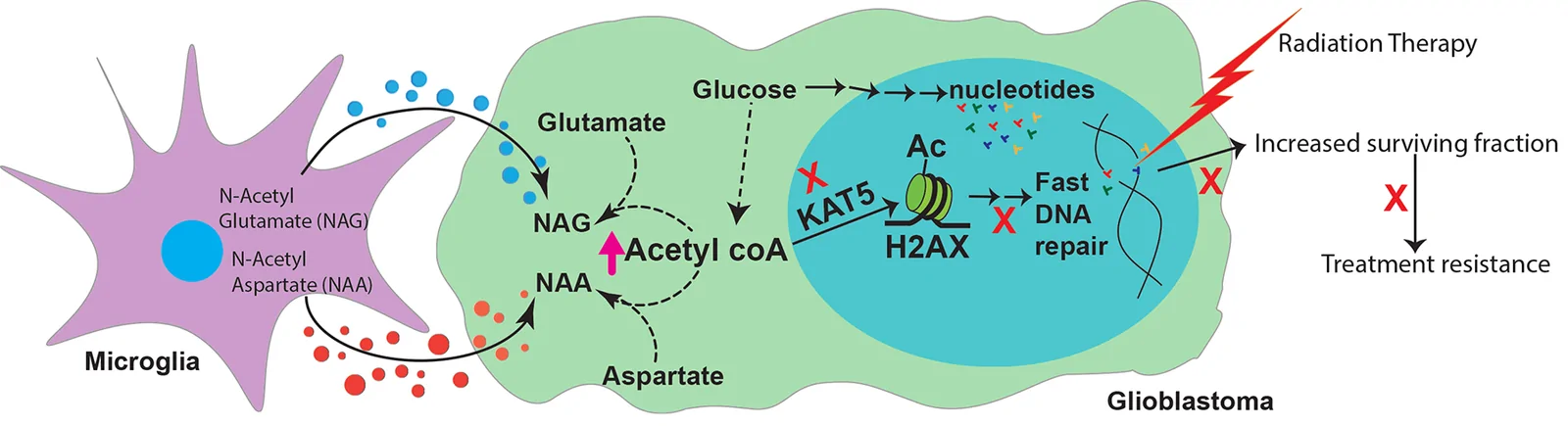
Schema. Cells in the microenvironment such as microglia secrete N-acetyl glutamate (NAG) and N-acetyl aspartate (NAA). These acetylated amino acids are taken up by glioma cells where they bolster metabolic activity leading to increased acetyl coA and nucleotide pools. This altered metabolism leads to heightened acetylation of histones following DNA damage thereby facilitating DNA repair and glioblastoma treatment resistance. Inhibiting the key histone acetyltransferase KAT5 in neoplastic glioblastoma cells reverses the protective effects of N-acetylated amino acids and microglia.

Altogether, our studies suggest that non-neoplastic cells such as microglia promote GBM RT resistance by supplying acetylated amino acids that bolster neoplastic cell metabolism and promote DNA repair via KAT5-mediated histone acetylation (Fig7).

## Discussion

In this study, we define mechanisms by which the intracranial glioblastoma microenvironment promotes DNA repair and treatment resistance through intercellular metabolite exchange (Fig 7). Across numerous models, the intracranial environment is associated with radiation resistance and rapid DNA repair. In laboratory models and human tumors, GBM neoplastic cell DNA repair is spatially heterogeneous with proximity to TAMs associated with more rapid resolution of DNA damage markers in neoplastic cells. Using reductionist laboratory models, we identified that the acetylated amino acids NAG and NAA are secreted by microglia and consumed by neoplastic GBM cells where they promote DNA repair and RT resistance. Using a variety of metabolomic approaches, we found that environmental NAG and NAA facilitate nucleotide synthesis and boost acetyl CoA pools in neoplastic cells, which supports histone acetylation and DNA repair. The protective effects of acetylated amino acids are lost when histone acetylation is impaired, suggesting a potential therapeutic strategy to overcome microenvironment mediated treatment resistance in GBM.

Our access to rare, acutely-irradiated human GBM tissues allowed us to interrogate neoplastic cell-intrinsic and -extrinsic regulators of DNA repair and RT resistance in the human condition. Glioma stem-like cells (GSCs) were previously shown to have enhanced DNA repair activity, which allow them to repopulate tumors following RT^38,39^. We found some support for this cell intrinsic regulation, as the two human GBM tumors resected shortly after RT (IRGBM1 and IRGBM2) showed lower γ-H2AX staining in GSC-like cells (Fig S1D), though associations between stemness and γ-H2AX were less clear in irradiated brain metastases or glioma explants irradiated *ex vivo*. By contrast, we did not observe associations between DNA repair and the recently described glioma cell states (Fig S1A–C), perhaps due to the plasticity of these states. By contrast, proximity of neoplastic cells to TAMs was associated with rapid DNA repair in neoplastic cells across patients and laboratory models, suggesting that neoplastic cell-extrinsic factors may be a critical regulator of GBM RT resistance. These factors may not be mutually exclusive, as GSCs can recruit TAMs, which can in turn influence neoplastic cells^71,72^. We are hopeful that the increasing use of post-treatment and on-treatment GBM biopsies^73^ will help bolster these analyses and allow a fuller understanding of how neoplastic cells respond to therapy.

Our observations across patient tissues and preclinical models linking TAMs, DNA repair and GBM radiation resistance fits well with previously published research. Indeed, RT can promote TAM infiltration into murine GBM, while TAM elimination can increase RT-induced DNA damage and improve treatment responses^9,74–76^. Similar associations are seen in human patients, where TAM infiltration and specific TAM subsets are associated with aggressive phenotypes and poor outcomes^77–79^. While eliminating specific TAM cell subsets could be a strategy to move forward beyond the disappointing clinical results of broad TAM depletion in GBM^80,81^, our studies and others^82,83^ suggest that interruption of metabolic communication between TAMs and neoplastic cells and downstream DNA repair could also be a promising treatment strategy.

This work also increases the understanding of how N-acetylated amino acids function in the brain tumor microenvironment^84^. NAA is one of the most abundant metabolites in the normal cortex and where it is canonically released by neurons and supports metabolism and myelination in oligodendrocytes^85^. Our work suggests that neoplastic glioma cells co-opt this process and utilize microenvironment-derived N-acetylated amino acids to fuel acetyl-coA and nucleotide synthesis, which in turn can promote histone acetylation, DNA repair and RT resistance. While our study focused on TAM-derived NAA, it is possible that tumors with increased neuronal content might utilize neuron-derived NAA similarly. Our human isotope tracing studies (Fig 3O) indicate active NAA (and NAG) synthesis in GBM tumors in patients. Thus, the low levels of NAA in GBM tumors may not just be due to low levels of NAA synthesis but rather may reflect increased uptake and utilization of NAA by neoplastic cells. This conjecture is strengthened by our NAA isotope tracing studies (S4U-W), showing that environmental NAA can contribute to aspartate and nucleotide pools in neoplastic glioma cells. Environmental NAG similarly bolsters neoplastic cell acetyl-coA and nucleotide pools. However, our isotope tracing studies suggest that it may do so by reducing synthetic demand of glutamate and acetyl-coA, rather than by direct use of NAG-derived carbon and nitrogen. Whether other N-acetylated molecules in the brain (such as N-acetyl-aspartyl-glutamate) plays a similar role in glioma biology remains to be discovered.

There are therapeutic implications to these discoveries. Interrupting neoplastic cell N-acetylated amino acid metabolism could slow GBM DNA repair and increase the efficacy of standard of care RT. However, the optimal strategy to target is uncertain. N-acetylated amino acids can come from numerous cell types, thus TAM depletion may not fully reverse this biology. Similarly, NAG and NAA are synthesized using different enzymes, have different downstream fates in neoplastic cells and have uncertain and likely redundant modes of uptake. However, our work suggests a common effect of heightened acetyl-coA levels and histone acetylation conferred by both NAG and NAA. Reversing these effects via KAT5/TIP60 inhibition blocked the ability of NAG, NAA and microglia to promote neoplastic cell DNA repair, suggesting that the KAT5/TIP60 inhibitors currently in development^86^ might be of use in GBM in combination with RT, especially if they can accumulate to active concentrations in the brain.

Our study also has limitations. We do not yet know the specific DNA repair pathways that are activated by NAG/NAA, though the heightened 53BP1 resolution and H2AX acetylation induction suggest that both NHEJ and HR might be affected. The extent to which different TAM subtypes secrete NAG and NAA is not known. We also do not know how and if NAG and NAA affect histone acetylation outside of the DNA repair context and thus it is possible that they confer additional aggressive phenotypes to GBM through other mechanisms such as epigenetic activation. Lastly, our acutely-irradiated GBM human tumor data came from only two patients. Though the data from these rare tissues was supported by consistent data from murine models, human brain metastases and *ex vivo* irradiated GBM explants, we are hopeful that analysis of additional patient tissues in the future can yield further insight into GBM treatment resistance.

In sum, this works describes a new type of metabolic communication between neoplastic cells and TAMs in the GBM microenvironment by co-opting known exchanges of N-acetylated amino acids. Given the many other known physiologic exchanges in the brain, we are hopeful that interrupting this and other as of yet undiscovered links could be a promising avenue to overcome GBM treatment resistance.

## Methods:

### Ethical Consideration

All mouse experimental procedures and handling performed in this study were approved by the Institutional Animal Care and Use Committee (IACUC) at the University of Michigan (Approval Number: PRO-00010680) and followed animal use guidelines put forth by the National Institute of Health (NIH). The use of human GBM tissues for this study was approved by the Institutional Review Board of the University of Michigan (Approval number: HUM00165469).

### Mouse models of GBM:

C57BL/6J mice aged 4–6 weeks obtained from Jackson Laboratories (Jackson 000664) were utilized in this study. Syngeneic mouse GBM tumors were generated using TRP cells which are mouse GBM cells derived from genetically engineered B6 mouse GBM tumors previously generated by and obtained from Dr. C. Ryan Miller^15,87,88^. TRP cells contain heterozygous TgGZT121 mutation downstream of the GFAP promoter, heterozygous KrasG12D mutation (R) and homozygous deletion of the tumor suppressor PTEN gene (P−/−). The TRP cells were then transduced with Green fluorescent protein (GFP) and firefly luciferase (Luc) reporter (pLenitLox CAG-dscGFP-T2A-Luc-EFS-Puro A, obtained from the University of Michigan Vector Core). These TRP cells expressing GFP and firefly luciferase were then injected into mouse flanks or orthotopically into the mouse brain as described previously^89^. The GFP enabled us to separate the tumor from adjacent normal tissue and the firefly luciferase enabled us to distinguish the injected neoplastic cells from the non-neoplastic cells in the microenvironment in subsequent single cell RNA sequencing analysis.

For study of DNA repair in flank tumors and orthotopic brain tumors, tumor-bearing mice were obtained after (i) no irradiation (n=3 each), (ii) within 20minutes after 4Gy irradiation (n=3 each) and (iii) at 6hrs after 4 Gy irradiation (n=3 each). These tissues were then fixed in 10% formalin and embedded in paraffin. The FFPE tissues were processed as described in immunofluorescence description.

### Single Cell RNA-Sequencing:

#### Sample Processing

Single cell suspension of TRP tumor tissue from a single mouse brain was generated and processed for library preparation and sequencing at the advanced genomics core, University of Michigan, following the Chromium Next GEM Automated Single Cell 3’ Reagent Kits v3.1 user guide (CG000286).

#### Single Cell RNA-Sequencing Data Processing

Single cell RNA-seq data was log-normalized and scaled using the Seurat pipeline (v5.3.1)^90^. Low quality cells were excused based on <500 or >10,000 detected genes, <1000 or >100,000 UMI counts, and > 12% mitochondrial transcripts. Cell type annotation of TRP tumor samples was done using *FindAllMarkers* function to determine cluster-specific marker genes. Luciferase expression (as mentioned above) was used to identify neoplastic cells. Marker based annotation of the clusters and subclusters was used to identify non-neoplastic cell types including myeloid cells, lymphoid cells, progenitor cells which did not express any advanced differentiation markers or luciferase and also to identify stem like subpopulations of the neoplastic cells.

### Patient tissue samples for spatial transcriptomics:

#### Unirradiated human GBM tissue for spatial transcriptomics (ST) on visium platform:

Under an institutional review board approved study in accordance with recognized ethical guidelines, intraoperative brain tumor specimens were collected at the University of Michigan using MRI-imaged guidance technology to localize specimen by co-senior author WNA. These specimens were obtained after written informed consent from patients. Eight Visium ST slides were profiled from three primary GBM patients. Slides 1512-A/B and 1512-C/D were collected from different portions of the same patient tumor. Slides 4774-A/B and slides 4774-C/D represent adjacent sections from two other patients.

#### Human GBM and metastatic lung cancer tissues for ST on visium (Cytassist) HD platform:

4 irradiated brain tumor sections with varying clinical and treatment conditions for brain tumor were obtained from patients and analyzed using Cytassist HD (Fig 2A):

1. Irradiated explant of human GBM tissue (GBM explant)- Glioblastoma tissue obtained during surgical resection and irradiated *ex vivo*.
2. Irradiated patient GBM 1 (IRGBM1)- Brain tumor tissue obtained from surgical resection 1 day post irradiation from a patient initially thought to have a brain metastasis from breast cancer and later diagnosed to be GBM.
3. Irradiated patient GBM 2 (IRGBM2)- Brain tumor tissue obtained from surgical resection 3 days post irradiation from a patient initially diagnosed to have a brain metastasis from lung cancer and later diagnosed to be GBM.
4. LungMet- Brain tumor tissue of obtained from a patient with metastatic non-small cell lung cancer obtained from surgical resection 1 day post irradiation.

Clinical details of these patients and molecular features of the tumors are described in the supplementary methods.

### Spatial transcriptomics:

#### Visium ST for unirradiated human GBM tissues:

##### Sample preparation:

Unirradiated Human GBM samples acquired were snap frozen in liquid nitrogen/isopentane followed by embedding in optimal cutting temperature compound. This tissue was sectioned and placed onto Visium Spatial Gene Expression Slide(10X Genomics) and further processed at the Advanced genomics core at the University of Michigan according to Visium spatial protocols (CG000240, CG000239). Sequencing data were analyzed with 10X genomics software(Space Ranger). The SpaceRanger output files were imported into R using the Load10x_Spatial function. QC processing is described in supplementary methods.

##### Visium Spot Deconvolution

Visium ST spot deconvolution was performed with *BayesPrism*^91^ using a comprehensive GBM single-cell atlas^92^ as reference. We used the level-3 annotation from the atlas for cell type cell state annotation. We downsampled the atlas to 3000 randomly selected cells per reference group. Raw counts for protein-coding genes from single-cell and Visium ST data were used to create the *BayesPrism* object. For the inference, we used *InstaPrism*^93^, which re-implements *BayesPrism* by replacing the Gibbs sampling step with a fixed-point algorithm, providing considerable speed and memory advantages.

##### Spot-level Scoring for DNA repair Gene Sets

KEGG genesets were programmatically downloaded using the msigdbR^94^ R package with settings species = ‘human’, collections = ‘C2’ and subcollections = ‘CP:KEGG_LEGACY’, and the dataframe filtered to only the five DNA repair pathways. Normalized counts per spot were used to compute enrichment scores for these genesets using the AddModuleScore^95^ function in Seurat using default settings.

##### Comparing Pathway activities with cell-type abundances

The inferred abundances of microglia and macrophages in each Visium spot is used to split the data across all eight slides into two categories for each cell-type. Spots with lower than 5% abundance are classified as having low infiltration, and vice versa. We then performed a t-test for activity scores for each DNA repair pathway versus the spot label (immune-low versus immune-high) to derive mean estimates per group and p-values. Violin plots for pathway scores between the two categories are generated using Seurat and edited with ggplot2^96^.

### **Visium Cytassist**:

#### Sample preparation:

Syngeneic mouse brain GBM (described above) tissues obtained (i) after no radiation exposure and (ii) 6hrs after RT (4 Gy) were fixed in 10% formalin and embedded in paraffin. The FFPE tissues were sectioned at 4 um thickness. These slides were then processed for IF for γ-H2AX, a marker for double stranded DNA breaks, using a protocol described in Visium Cytassist for FFPE tissue preparation handbook (CG00519) with the minor modification of using 0.2% Triton X to enhance permeabilization of the tissue. Whole slide scanning to obtain fluorescent microscopy images of the entire tissue sections at 20x magnification was performed on Cytation 5 (Biotek) imager. Following imaging the sections were processed for probe-based library construction and sequencing at the Advanced Genomics core, University of Michigan according to the Visium CytAssist Spatial Gene Expression Reagent Kits User Guide (CG000495). The data was analyzed using space ranger software.

#### Spatial Deconvolution

Deconvolution of Visium 50 um spots was performed using the *BayesPrism* and *InstaPrism* algorithm^91,93^. The curated single-cell RNA-seq data for TRP tumor sample was used as the reference in the algorithm to create the *BayesPrism* object. The fractions of each cell type for each spot were saved in the Seurat object. A threshold of spots containing 8% fraction of microglia and macrophages were used to compare between GH2AX-positive and -negative spots.

### **Visium Cytassist HD**:

#### Sample preparation:

FFPE tissue sections from GBM explant, IRGBM1, IRGBM2 and LungMet tissues described above were processed for IF for γ-H2AX using a protocol described in Visium Cytassist for FFPE tissue preparation handbook (CG00519) with modifications to the antigen retrieval temperature (80°C for 30 min decrosslinking) to allow for gentler processing required for HD spatial transcriptomics and including a 0.2% triton X treatment as above. Whole slide scanning to obtain fluorescent microscopy images of the entire tissue sections at 20x magnification was performed on Cytation 5 (Biotek) imager. Following imaging the sections were processed for probe-based library construction and sequencing at the Advanced Genomics core following the Visium HD Spatial Gene Expression Reagent Kits User Guide (CG000685). Cellpose version 2.0 and Space Ranger version 3.0 were used for analysis.

#### High sensitivity nuclear segmentation and preprocessing

To achieve single cell resolution across dense tumor regions, DAPI channel images were preprocessed using Contrast Limited Adaptive Histogram Equalization (CLAHE) with a 2.0 clip limit to enhance nuclear boundaries. Automated nuclear segmentation was performed using Cellpose (version 2.0)^97^ with a GPU accelerated “nuclei” model. To ensure the capture of small or faint nuclei within the complex tumor microenvironment of irradiated samples, a unified high sensitivity configuration was implemented across all four samples by setting the *cellprob_threshold* to −2.0 and the *flow_threshold* to 0.6. The resulting nuclear masks were exported as TIFF files for integration with the spatial transcriptomic pipeline.

#### Custom segmented spatial transcriptomics pipeline

To generate cell-centric transcriptomic profiles, the Cellpose derived nuclear masks were integrated into the 10x Genomics Space Ranger HD (v4.0.1)^98^ pipeline. For each sample (GBM explant, LungMet, Irradiated patient GBM1 (IRGBM1) and Irradiated patient GBM2(IRGBM2), the analysis was executed using the *spaceranger count* command with the –*custom-segmentation-file* parameter. To ensure high fidelity cell capture and minimize the loss of cells due to minor alignment variations or boundary errors, a 2-micron expansion distance was applied to the nuclear masks (--*nucleus-expansion-distance-micron*=2). This expansion served as a computational buffer, increasing the sensitivity of the transcript to cell assignment and ensuring that nuclei were not excluded due to stringent boundary constraints. Raw sequencing reads were aligned to the GRCh38 (2024-A) reference genome^99^, resulting in feature-barcode matrices where transcript counts were aggregated within the biologically informed and computationally buffered boundaries of individual detected cells.

#### Quantification of DNA damage and signal filtering

Following the generation of custom segmented spatial matrices, DNA damage levels were quantified by extracting immunofluorescence signal intensities for the γ-H2AX (Red) and DAPI (Blue) channels within each segmented nuclear boundary. Using specialized thresholding strategies described in the supplementary methods, the cells were classified as γ-H2AX high (damage high) and γ-H2AX low (damage low) cells. These classifications were integrated into the Seurat (v5.4.0)^90^ metadata as a binary “DNA damage status” (Damage-High vs Damage-Low) for downstream spatial analysis.

#### Reference-based cell type annotation

To biologically annotate the custom segmented cells, supervised label transfer was performed using two independent glioblastoma single cell RNA-sequencing (scRNA-seq) references. The primary reference comprised a large scale atlas of human glioblastoma consisting of 123,236 cells sampled from the tumor core to the macroscopically normal cortex^28^. A secondary reference was utilized to further refine cellular identities, including neoplastic states and microenvironmental populations^100^. The transfer workflow was implemented using the Seurat (v5.1.0)^90^ anchor-based framework. An RNA assay was generated for each Visium HD object by extracting raw counts from the custom segmented nuclear boundaries. Both query and reference datasets were log normalized, and the top 2,000 highly variable features were identified. Transfer anchors were identified via Canonical Correlation Analysis (CCA) using the *FindTransferAnchors* function, projecting the datasets into a shared low dimensional space based on the first 30 dimensions. Cell type labels were projected onto the spatial datasets using the *TransferData* function. To correct for potential mislabeling of cells due to overlap stemming from nuclear expansion based segmentation, marker based confirmation of annotation was performed using *AddModuleScore* function for expression of canonical markers for the different cell types identified. For each segmented cell, a maximum prediction score and a full probability matrix were calculated, and only cells with high confidence predictions were utilized for downstream analysis.

#### Malignant state and lineage hierarchy analysis

To characterize the phenotypic diversity of neoplastic cells, relative lineage and differentiation scores were calculated based on established glioblastoma cellular states (AC, MES, NPC, OPC) as describe previously^37^. Briefly, for each custom segmented cell, prediction scores for these four states were extracted from the reference based annotation and assigned quadrant coordinates. These coordinates were used to project neoplastic cells into a 2D hierarchy, often referred to as a “butterfly” plot to visualize state shifts associated with DNA damage^37^. This is described in detail in supplementary methods.

#### Spatial neighborhood and non-neoplastic cell count analysis

To characterize the spatial relationship between neoplastic DNA damage and the non-neoplastic cells in the microenvironment, a localized neighborhood analysis was performed. To quantify non-neoplastic cell content in these specific tumor niches, a **50 μm** radius was defined around each neoplastic cell. The number of neighboring non-neoplastic cells within this radius was calculated using a *k-nearest neighbor* search (*k* = 100) via the *nabor* package^101^.

The radius for the tumor niches was defined after analyzing the cell counts present within circular regions around index neoplastic cells with radii ranging from 25μm to 300μm in 2 of the tissue sections. As seen in the table below, 50 μm radius was the smallest neighborhood containing at least 1 neighboring cell on an average in both the tissues.

Despite the small size of the neighborhoods, >90% of the neighborhoods overlapped in all the tissues studied, necessitating the identification of non-overlapping “Zones” containing clusters of γ-H2AX positive and γ-H2AX negative neoplastic neighborhoods.

To achieve this, spatial coordinates were embedded into a two dimensional map, and a distance-based weight matrix was constructed. Spatial “hotspots” of tumoral DNA damage were identified by calculating the local Getis-Ord $G_{i}^{*}$ statistic for each neoplastic cell based on its γ-H2AX status using the *spdep* package^40^. By focusing the $G_{i}^{*}$ calculation on the neoplastic compartment, cells were categorized into “Slow repair zones” (neighborhoods with high densities of γ-H2AX positive neoplastic cells; $G_{i}^{*}>1.645$), “Fast repair zones” (neighborhoods with low densities of γ-H2AX positive neoplastic cells; $G_{i}^{*}<1.645$), or “Transition” zones.

Differences in non-neoplastic cell content between “Fast repair zones” and “Slow repair zones” were evaluated using Wilcoxon rank-sum tests.

#### Differential expression and pathway enrichment

Differential expression (DE) was performed using the *FindMarkers* function in Seurat (v5.4.0)^90^ across two primary axes:

1. Global DNA damage status (Damage-High vs. Damage-Low) and
2. Spatial Zonal status (Fast repair vs. Slow repair).

These comparisons were executed for the total neoplastic population, individual Neftel states, and myeloid populations. Genes with an *adjusted p-value* < 0.05 and |*avg*_*log*_2_*FC*| > 0.1 were considered significant.

#### Gene Ontology analysis:

Commonly enriched transporter genes in TAM subpopulations within the fast repair and slow repair zones in different samples and commonly enriched genes in γ-H2AX negative (damage low) neoplastic cells in IRGBM1 and IRGBM2 were analyzed for pathway enrichment using Enrichr web tool and output graphs were generated using Appyter.

### Immunofluorescence (IF):

#### 1. Multiplex IF:

FFPE sections from Irradiated brain TRP tumor and irradiated GBM explant tissues described above were used to study TAM abundance in fast DNA repair regions using an orthogonal method. TAMs were identified based on protein expression for IBA1 and γ-H2AX immunofluorescence was used to identify DNA damage in the cells

Multiplex IF staining was performed using the Opal protocol, as described previously^102^. Briefly, the staining protocol for each individual primary antibody was first optimized and each of the sections mentioned above were sequentially stained with a panel of antibodies (Supplementary methods, Table1) using a Ventana Discovery Ultra stainer (Ventana Medical Systems). After incubation with the primary antibody followed by a Tris-buffered saline with Tween-20 buffer (TBST) wash, Ventana OmniMap anti-rabbit horseradish peroxidase followed by Opal Tyramide Signal Amplification were applied. This creates a covalent bond between the tissue and the fluorophore at the horseradish peroxidase site. Following this, heat-induced antigen retrieval was performed using CC2 buffer to remove the primary antibody/secondary antibody complex, and the next antibody was applied. A panel of 6 fluorochromes was sequentially applied to each tissue section. Only 2 of them (IBA1 and γ-H2AX) were analyzed for this study. The slides were then mounted with ProLong Gold containing DAPI and scanned using an Akoya Polaris IF scanner at 20× magnification. The images were visualized on Phenochart to perform spectral unmixing and exported to qupath v0.6.0. for neighborhood analysis.

A custom Groovy script was developed in QuPath to automatically identify local γ-H2AX-positive and γ-H2AX-negative regions within tumor tissues. The script scanned each tumor tissue using circular regions of interest (ROIs) whose radii were optimized based on cell count within the ROI. A mean count of 50 cells per ROI were identified within a radius of 100μm in the TRP tissue and a radius of 150μm in GBM explant tissue. For each ROI, the total number of cells, the percentage of γ-H2AX-positive, IBA1 negative cells, and the percentage of Iba1-positive cells. ROIs with >10% γ-H2AX-positive cell were classified as γ-H2AX-positive regions, whereas ROIs with <10% γ-H2AX-positive cell fractions were classified as γ-H2AX-negative regions. The proportion of TAM-enriched ROIs was compared between γ-H2AX-positive and γ-H2AX-negative neighborhoods using Fisher’s exact test. For TRP tissue, a threshold of 50% TAM abundance was used to define TAM enriched ROIs. For the GBM explant, given the relative sparsity of tissue, we defined TAM enriched ROIs using a threshold of 25% TAM abundance.

#### 2. Immunofluorescence for γ-H2AX and 53BP1:

Monocultures and cocultures of GBM cells and microglia were grown on coverslips as described above and irradiated at 4 Gy. The cells were fixed using 4% Paraformaldehyde, at 3 different time points post-irradiation: 20 minutes, 2 hours, and 6 hours.

FFPE tissues obtained from TRP flank and brain tumors obtained as described above were sectioned at 4um thickness onto charged glass slides.

Immunoflurescence for was performed as described in the supplementary methods. Briefly, FFPE tissues were deparaffinized and subjected to antigen retrieval followed by permeabilization. Cells on coverslips were fixed and permeabilized. This was followed by blocking, incubation with primary antibody and a fluorescent (Texas red) secondary antibody with DAPI counterstaining.

Fluorescent images were captured using the BioTek Cytation 5 confocal fluorescence microscope. For each replicate, three representative fields were imaged and analyzed using Gen5 software (version 3.12), as described in the supplementary methods. Briefly, the images were obtained at 20x and segmented using DAPI to identify cells. The no. of Texas red foci per nucleus and the average intensity of Texas red per nucleus were measured using the Gen5 cytation software. A threshold of 20 foci and a threshold of Texas red intensity established based on a positive and negative control for each experiment were used to identify cells positive for γ-H2AX or 53BP1.

### Cell Culture:

Human GBM cell lines, GBM38 and GBM6 obtained from the Dr. Jann Sarkaria lab from the Patient-Derived Xenograft (PDX) National Resource at the Mayo clinic. Immortalized human microglia (HMC3 cells) were purchased from the American Type Culture Collection (ATCC, catalog No. CRL-3304). HPM, or human primary microglia, used in this study were obtained from Celprogen (Catalog No. 37089–01). As mentioned above, TRP cells are murine GBM cells originating from genetically engineered GBM derived from C57/BL6 mouse. The cells were cultured as monocultures as described in the supplementary methods.

#### Supplementation of N-acetyl-L-glutamate (NAG) and N-Acetyl-Aspartate (NAA)

GBM38 and GBM6 cells received 100μM N-acetyl glutamate (NAG) (Tokyo Chemical Industry, Product No. A0693, MW = 189.17g/mole) or 1mM NAA (Cayman Chemical, Item No. 34635). - Unless otherwise specified, cells were treated with NAG or NAA for 48 hours prior to experimental assays. Media was replenished with NAG immediately before radiation treatments when experiments included it.

#### Microglia Coculture

Equal numbers of GBM cells(wildtype or sgKAT5 cells) and microglia were plated either in contact or separated by a cell culture-treated insert (Corning Inc., 12 well inserts cat no.353181, Cellquart 6 well-culture inserts, cat no. 930 04 12). For contact coculture, GBM cells were first fluorescently labelled with a Green (emission at 488nm) fluorescent membrane dye (Celltrace CSFE, cat no. C34570, Thermofisher) and then cocultured with non-fluorescent microglia, plated together into 12-well plates. For a contactless coculture, the GBM cells were cultured in the wells of a 12- well plate or a 6 well plate, while the cell culture-treated inserts contained the microglia. The cells were cocultured for at least 48hrs prior to experimental irradiation or sample processing for metabolomics.

### Experimental Irradiation:

Irradiation for experiments were performed in the Experimental Irradiation Shared Resource using an IC-225 Orthovoltage machine (Kimtron Medical). Radiation doses for the experiments are indicated in the figure legends.

### Targeted Metabolomics:

Conditioned media from monocultures, cocultures, as well as control media were collected as 80% methanol solutions and processed for metabolite extraction. Intracellular metabolites were analyzed in GBM cells subjected to different experimental conditions, as described in the supplementary methods. Briefly, the cells were lysed using 80% methanol and processed for metabolite extraction as described previously^5^. Briefly, the 80% methanol lysates/solutions were centrifuged to pellet insoluble materials. The supernatants were collected and blow-dried using the nitrogen blower. Dried metabolite pellets were resuspended in 50% methanol and subjected to LC-MS as described below.

#### LC-MS and data analysis:

Samples obtained from the conditioned media experiment and the intracellular metabolite samples obtained from the targeted metabolomics NAG or NAA supplementation assays were analyzed using an Agilent Technologies Triple Quad 6470 liquid chromatography–tandem mass spectrometry system, with the 1290 Infinity II LC flexible pump (Quaternary Pump), the 1290 Infinity II multisampler, the 1290 Infinity II multicolumn thermostat with six-port valve and the 6470 triple quad mass spectrometer, as described previously^6^. The LC/MS data acquisition and compound optimization were performed using Agilent MassHunter Workstation software for 6400 Series Triple Quadrupole MS with version B.08.02. Studies were performed in negative ion acquisition mode with ion-pairing chromatography using an Agilent ZORBAX RRHD Extend-C18, 2.1 × 150 mm, 1.8 μm and ZORBAX Extend Fast Guards for UHPLC separation. Metabolite peaks were integrated and peak areas were quantified using Agilent MassHunter Workstation quantitative analysis for QQQ version 10.1, build 10.1.733.0. Peak abundances were normalized to the cell number or protein concentration across all the samples being compared. The normalized data was used to generate heatmaps on Morpheus.

### Stable Isotope Tracing:

Stable isotope tracing experiments were conducted using either ^13^C_6_ Glucose (all 6 carbons labelled; Cambridge Isotope Laboratories, CLM-1396–1), ^13^C_5_ N-Acetyl-Glutamate (5 Glutamate carbons labeled and 2 acetate carbons unlabeled; Cambridge Isotope Laboratories, CLM-6664), ^15^N-ammonium (Cambridge Isotope Laboratories, Catalog No. NLM-467–1) or ^13^C_4_-labeled N-acetyl aspartate (4 aspartate carbons labeled and 2 acetate carbons unlabeled; Sigma-Aldrich, cat no. 683647). Dialyzed FBS (One shot, Invitrogen, catalogue no. A3382001) was used in all tracing conditions.

Experimental conditions for ^15^N-ammonium tracing, ^13^C_5_ NAG tracing and ^13^C_4_ NAA tracing are described in the supplementary methods.

#### ^13^C_6_ Glucose tracing:

##### GBM monocultures:

GBM cells were cultured in ^13^C_6_ Glucose labeled media with (treatment group) or without (control group) NAG or NAA supplementation. Unlabeled glucose supplemented cells served as tracing negative controls. Glucose-free DMEM (Invitrogen, 11966025) supplemented with 4.5 g/L ^13^C_6_-glucose was used for the labeled condition.

##### Coculture before cell sorting by flow cytometry:

HMC3 microglia were cultured in ^13^C_6_ Glucose labeled media for 1 week and fluorescently marked with a fluorescent (emission at 405/450) membrane dye (Celltrace violet dye, C34571, Thermofisher). These cells were then cocultured with GBM cells in complete media lacking ^13^C_6_ Glucose overnight. The cells were resuspended into single cells then sorted using flow cytometry on SonySH800 instrument, using the fluorescent celltrace violet marker on the microglia for gating. The sorted cells were snap frozen in LN_2_ and stored at −80 until further processing for metabolite extraction.

Metabolites were extracted from all the cells as described above, using 80% methanol followed by precipitation of insoluble materials, and blow drying the soluble fraction with Nitrogen and resuspending with 50% methanol.

### Human stable isotope tracing with ^13^C_6_ Glucose:

The human isotope tracing samples analyzed in this study were obtained with approval by the Institutional Review Board of the University of Michigan. After obtaining informed consent, patients with suspected GBM were enrolled onto our clinical study, as described previously^5^. Briefly, patients received a bolus intravenous dose of [U^13^C] glucose (8 g) followed by a continuous intravenous infusion of [U^13^C] glucose at a rate of 4 g h^−1^. After craniotomy and tumor exposure, the surgeon identified radiographically defined enhancing tumor and adjacent cortical tissues using stereotactic image guidance. These tissues were then resected and separated by a board-certified neurosurgeon (W.N.A.-H.), rinsed in cold PBS and immediately (typically less than 3 min after resection) flash-frozen in liquid nitrogen for further analysis. Tumor diagnosis and molecular information including IDH mutation status were abstracted from the medical records and clinical pathology report. Only the tumors with confirmed diagnosis of Glioblastoma, IDH wildtype were included in this study. A total of eight tissues met the inclusion criteria. Five of them were from our prior study^5^ while three more were collected more recently. The two sets of tissues were analyzed on separate mass spectrometers. The previously published data set was processed on an Agilent QTOF mass spectrometer while the three recent tissues were processed on Orbitrap IQ-X Tribrid mass spectrometer, as described below. To correct for technical differences, the total abundance fold changes were calculated in the tumors compared to the cortex samples obtained from the same patient and processed on the same instrument. These fold changes were then compared across samples.

### LC-MS for detection of labelled isotopes:

Isotope labelling for ^13^C_6_ Glucose tracing, ^13^C_5_ NAG tracing and ^13^C_4_ NAA tracing was determined using an Agilent system consisting of an Infinity Lab II UPLC coupled with either a 6545 or 6230 QTOF mass spectrometer (Agilent Technologies) operated by the University of Michigan Metabolomics Core, as described before^5^. The data were analyzed with values corrected for natural isotope abundance using MassHunter Profinder 10.0. Samples without ^13^C labelling were used as tracing controls to ensure that the detected labelled isotopologues were not from contaminating species.

For ^15^N-ammonium stable isotope tracing, we used previously described methods by our team^103^. A Thermo Scientific Orbitrap IQ-X Tribrid mass spectrometer using an injection volume of 3 μL was used to detect ^15^N isotope labeling. The Waters ACQUITY UPLC BEH Amide 2.1 × 100 mm, 1.7μm column (186004801) was used. The column was held at 30 °C; the flow rate was 0.2 ml/min. Mobile Phase A was compised of 0.1% Ammonium hydroxide in purified deionized water with 20 mM Ammonium carbonate; Mobile Phase B was comprised of pure acetonitrile. Each sample was subjected to a linear gradient as follows: 0–0.5 min 85% B, 0.5–15 min 15% B, 15–17 min 15% B, 17–17.1 min 85% B, 17.1–22 min 85% B.

Peak areas were extracted in Skyline and manually integrated using an in-house list of standard m/z and retention times for known metabolites. Natural isotope abundance correction was performed with IsocorrectoR.

### Acetyl coA assay:

To assess the effect of NAG or NAA supplementation on intracellular acetyl coa levels, we compared GBM cells cultured in complete media with GBM cells supplemented with 100uM NAG, 1mM NAA, 4M sodium acetate (Sigma, catalogue no. S2889) for 48hrs or subjected to overnight glucose starvation using Glucose free DMEM with 10% dialyzed FBS and 1% penstrep. The sodium acetate supplemented cells served as positive controls and the glucose starved condition served as negative control. The assay principle is described in the supplementary methods. The NADH generated during the conversion of acetylcoA in the sample to citrate was measured at 340nm on Cytation 3 (Biotek) microplate reader.

Clonogenic Survival Assay, CRISPR/Cas9 gene knockdown and western blot analysis are described in supplementary methods.

## Supplementary Material

1

## Figures and Tables

**Table1.** Mean non-neoplastic cell counts in neoplastic cell neighborhoods with different radii.

GBM explant		LungMet	
radius	Mean neighbors	radius	Mean neighbors
25	0.537867	25	0.33328
50	3.563056	50	2.734784
75	8.58707	75	6.435588
100	15.3864	100	11.50254
125	24.03123	125	17.8563
150	34.51889	150	25.46909
175	46.62082	175	34.40563
200	60.48799	200	44.56039
225	75.88329	225	56.04472
250	92.99882	250	68.71181
275	111.4715	275	82.68096
300	131.4544	300	97.86837

## Data Availability

Single cell RNAseq data for the TRP tumor are publicly available for download and visualization via the Single Cell Portal: SCP3334 (TRP). Spatial transcriptomics data for unirradiated human tissue- The SC and ST data generated in this study are provided as Python AnnData objects through the following link (https://zenodo.org/records/16505469). Spatial transcriptomics data for mouse GBM and HD spatial transcriptomics data of irradiated human GBM and LungMet- The data will be made publicly available upon acceptance.
