# Supplementary material for: Microenvironment-derived acetylated amino acids promote glioblastoma treatment resistance
Source: Res Sq. 2026 Sep 18:rs.3.rs-10976328. Preprint. [Version 1] doi: 10.21203/rs.3.rs-10976328/v1 (PMC13596666; doi:10.21203/rs.3.rs-10976328/v1)
Supplement: Supplementary file 1 [file NIHPPRS10976328V1-supplement-1.pdf]

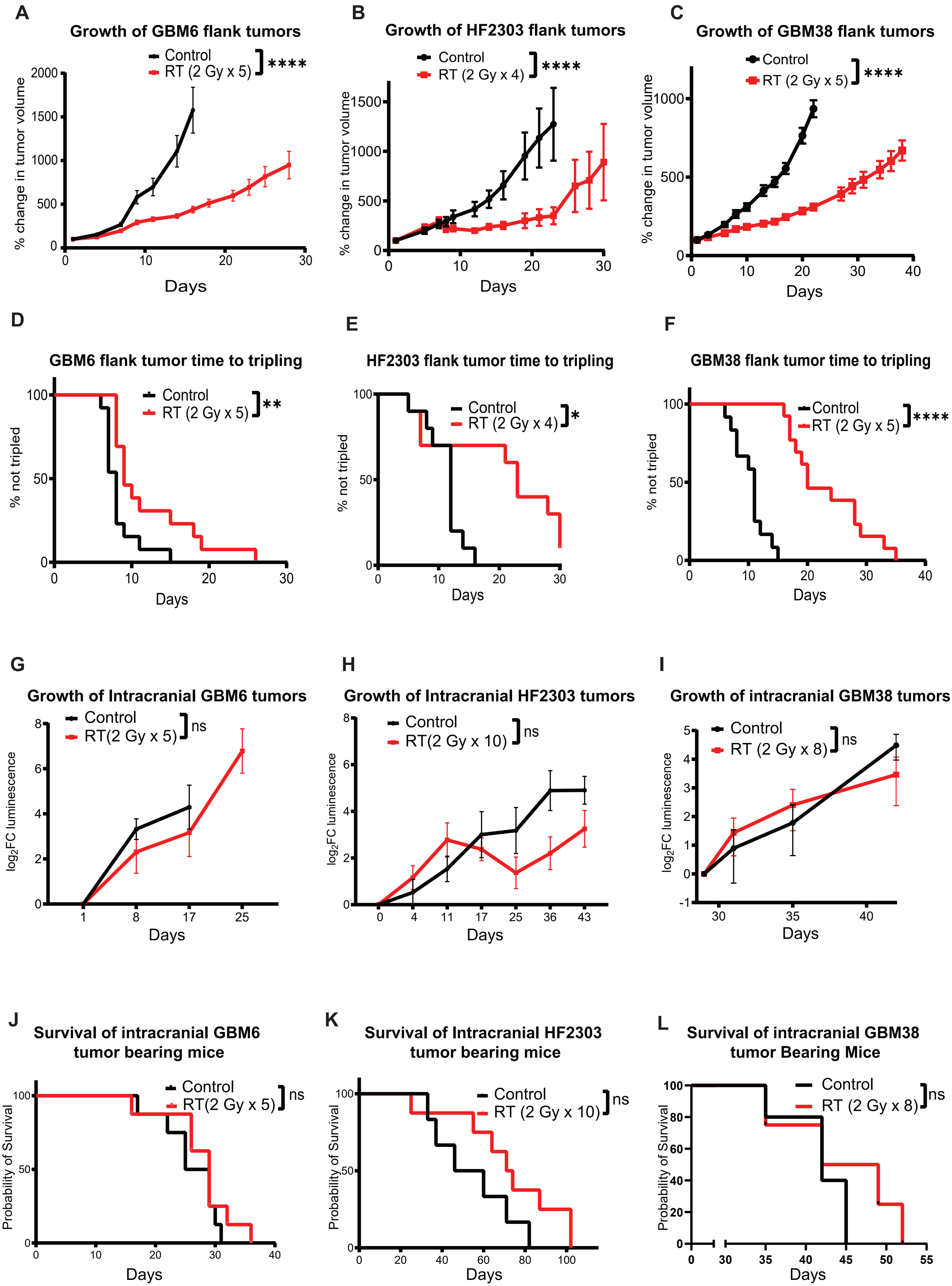
A
Growth of GBM6 flank tumors
Control
RT (2 Gy x 5)
****
% change in tumor volume
Days
B
Growth of HF2303 flank tumors
Control
RT (2 Gy x 4)
****
% change in tumor volume
Days
C
Growth of GBM38 flank tumors
Control
RT (2 Gy x 5)
****
% change in tumor volume
Days
D
GBM6 flank tumor time to tripling
Control
RT (2 Gy x 5)
**
% not tripled
Days
E
HF2303 flank tumor time to tripling
Control
RT (2 Gy x 4)
*
% not tripled
Days
F
GBM38 flank tumor time to tripling
Control
RT (2 Gy x 5)
****
% not tripled
Days
G
Growth of Intracranial GBM6 tumors
Control
RT(2 Gy x 5)
ns
log2FC luminescence
Days
H
Growth of Intracranial HF2303 tumors
Control
RT(2 Gy x 10)
ns
log2FC luminescence
Days
I
Growth of intracranial GBM38 tumors
Control
RT (2 Gy x 8)
ns
log2FC luminescence
Days
J
Survival of intracranial GBM6 tumor bearing mice
Control
RT(2 Gy x 5)
ns
Probability of Survival
Days
K
Survival of Intracranial HF2303 tumor bearing mice
Control
RT (2 Gy x 10)
ns
Probability of Survival
Days
L
Survival of intracranial GBM38 tumor Bearing Mice
Control
RT (2 Gy x 8)
ns
Probability of Survival
Days

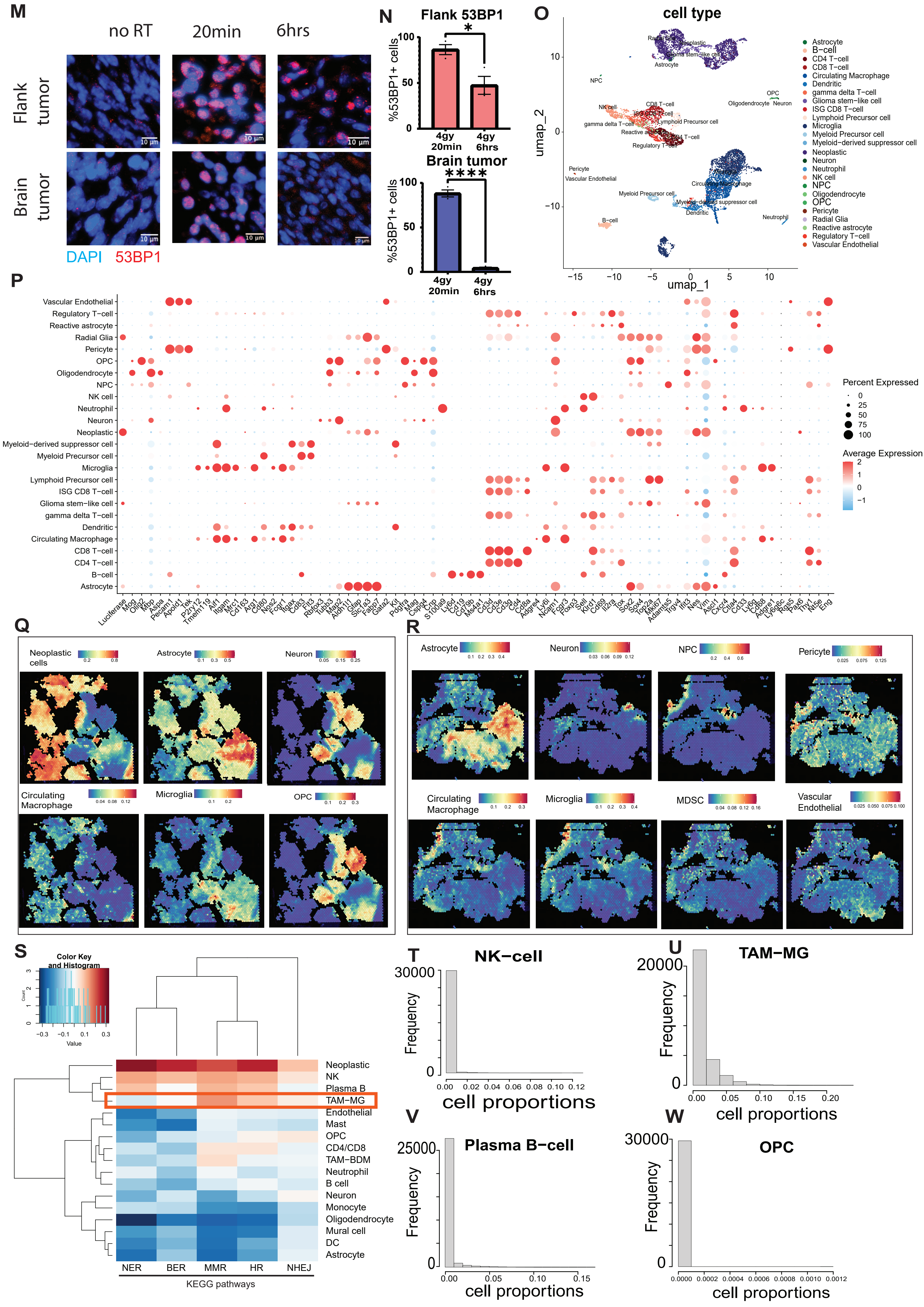

M
no RT
20min
6hrs
Flank tumor
Brain tumor
10 µm
DAPI
53BP1
N
Flank 53BP1
%53BP1+ cells
4gy 20min
4gy 6hrs
Brain tumor
O
cell type
umap_1
umap_2
Astrocyte
B-cell
CD4 T-cell
CD8 T-cell
Circulating Macrophage
Dendritic
gamma delta T-cell
Glioma stem-like cell
ISG CD8 T-cell
Lymphoid Precursor cell
Microglia
Myeloid Precursor cell
Myeloid-derived suppressor cell
Neoplastic
Neuron
Neutrophil
NK cell
NPC
Oligodendrocyte
OPC
Pericyte
Radial Glia
Reactive astrocyte
Regulatory T-cell
Vascular Endothelial
P
Percent Expressed
Average Expression
Vascular Endothelial
Regulatory T-cell
Reactive astrocyte
Radial Glia
Pericyte
OPC
Oligodendrocyte
NPC
NK cell
Neutrophil
Neuron
Neoplastic
Myeloid-derived suppressor cell
Myeloid Precursor cell
Microglia
Lymphoid Precursor cell
ISG CD8 T-cell
Glioma stem-like cell
gamma delta T-cell
Dendritic
Circulating Macrophage
CD8 T-cell
CD4 T-cell
B-cell
Astrocyte
Q
Neoplastic cells
Astrocyte
Neuron
Circulating Macrophage
Microglia
OPC
R
Astrocyte
Neuron
NPC
Pericyte
Circulating Macrophage
Microglia
MDSC
Vascular Endothelial
S
Color Key and Histogram
Count
Value
Neoplastic
NK
Plasma B
TAM-MG
Endothelial
Mast
OPC
CD4/CD8
TAM-BDM
Neutrophil
B cell
Neuron
Monocyte
Oligodendrocyte
Mural cell
DC
Astrocyte
NER
BER
MMR
HR
NHEJ
KEGG pathways
T
NK-cell
Frequency
cell proportions
U
TAM-MG
V
Plasma B-cell
W
OPC

**Supplementary figS1**. **A-C.** Percent change in flank tumor volume of (A) GBM6(n=13 tumors in each condition), (B) HF2303(n=10 tumors in each condition) and (C) GBM38 (n=12 tumors in control and 13 tumors in RT condition) PDX models. **D-F.** Kaplan Meier curves depicting time to tumor tripling in (D) GBM6, (E) HF2303 and (F) GBM 38 flank tumors **G-I.** Intracranial tumor volume, calculated as the log fold change in luminescence of the luciferase reporter carrying PDX tumor models (G) GBM6(n=8 mice in each condition), (H) HF2303(n=6 mice in control and 8 mice in RT condition), (I) GBM38(n=5 mice in control and 4 mice in RT condition). **J-L.** Kaplan Meier survival curves depicting overall survival of intracranial PDX tumor bearing mice. **A-L** are abstracted and replotted from our prior publications[3,14]. P values for tumor growth curves were calculated using Two-way Anova and indicated as * 0.01 to 0.05, ** 0.001 to 0.01, *** 0.0001 to 0.001, ****<0.0001, ns >0.05.All error bars indicate SEM **M.** Representative images of 53BP1 immunofluorescence in flank and brain tumors after 4 Gy RT. Scale bar 10µm. **N.** Quantification of % 53BP1$^+$ cells at 20 min and 6 hrs following RT (4 Gy) showing lesser remaining damage at 6 hrs in intracranial tumors (n=3 tumors per condition). P-value calculated using unpaired T-test. **O.** UMAP of single cell RNA sequencing data from TRP tumors showing annotation of different cell types. **P.** Dotplot showing markers used for marker-based annotation of the different cell types of the TRP tumor. **Q.** Spatial map of unirradiated, orthotopic TRP brain tumor showing spatial distribution of neoplastic cells and non-malignant cell types with >1% mean fractional abundance in the tumor. **R.** Spatial map of irradiated, orthotopic TRP brain tumor showing spatial distribution of non-malignant cell types with >1% mean fractional abundance in the tumor. **S.** Correlation heatmap showing cell type and DNA repair pathway activity (identified using Kyoto Encyclopedia of Genes and Genomes (KEGG) pathway analysis) in human unirradiated GBM tissues. **T-W.** Histograms showing abundance of other cell types partly associated with higher DNA repair pathway activity, suggesting that microglia (TAM-MG) are the most abundant cell type associated with higher baseline DNA repair pathway activity in human GBM.

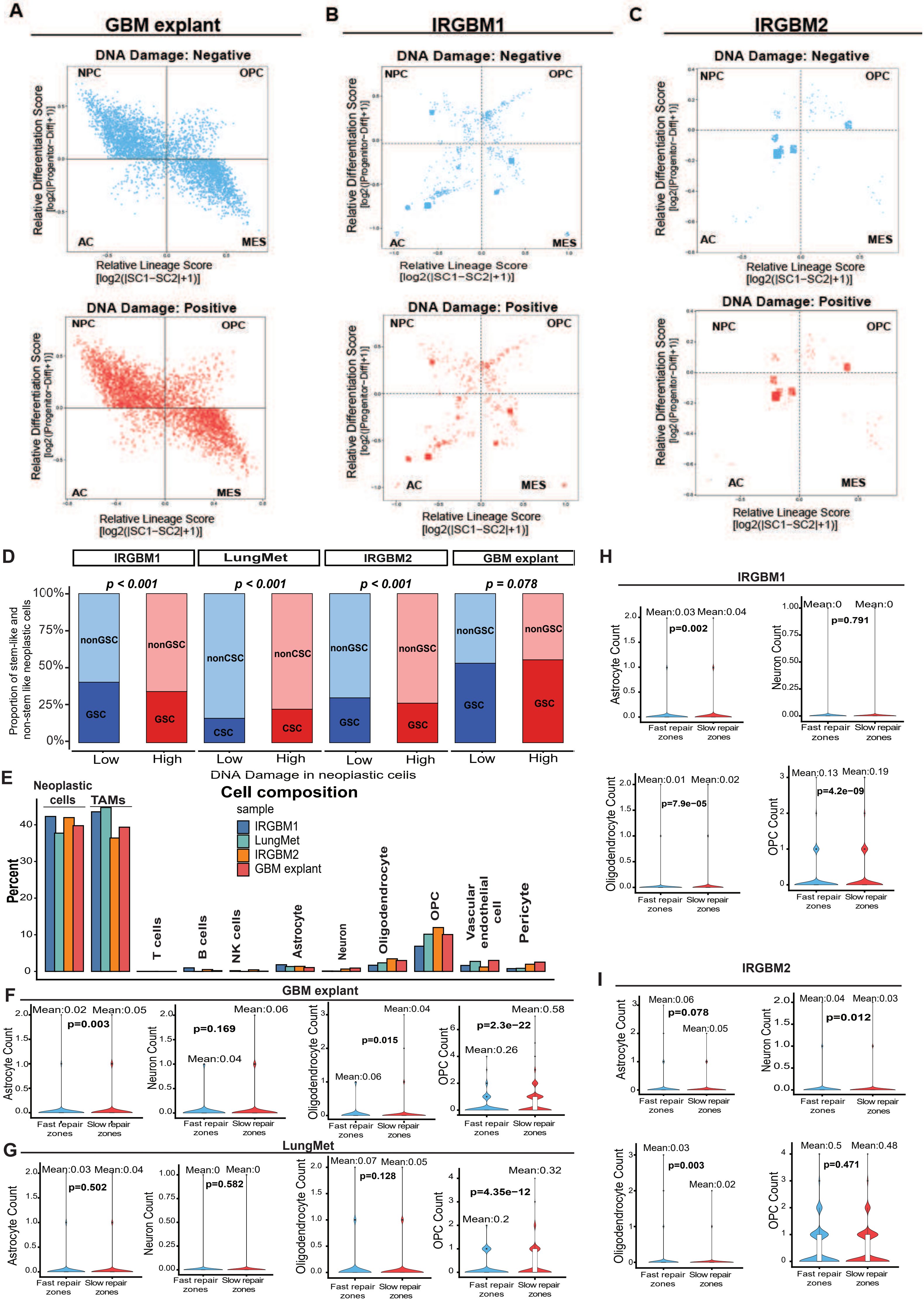

A
GBM explant
B
IRGBM1
C
IRGBM2
DNA Damage: Negative
DNA Damage: Positive
NPC
OPC
AC
MES
Relative Differentiation Score
[log2(|Progenitor−Diff|+1)]
Relative Lineage Score
[log2(|SC1−SC2|+1)]
D
IRGBM1
LungMet
IRGBM2
GBM explant
p < 0.001
p < 0.001
p < 0.001
p = 0.078
Proportion of stem-like and non-stem like neoplastic cells
nonGSC
GSC
nonCSC
CSC
Low
High
DNA Damage in neoplastic cells
E
Cell composition
sample
IRGBM1
LungMet
IRGBM2
GBM explant
Percent
Neoplastic cells
TAMs
T cells
B cells
NK cells
Astrocyte
Neuron
Oligodendrocyte
OPC
Vascular endothelial cell
Pericyte
F
GBM explant
G
LungMet
H
IRGBM1
I
IRGBM2
Astrocyte Count
Neuron Count
Oligodendrocyte Count
OPC Count
Fast repair zones
Slow repair zones
Mean:0.02 Mean:0.05 p=0.003
p=0.169 Mean:0.04 Mean:0.06
Mean:0.06 Mean:0.04 p=0.015
Mean:0.26 Mean:0.58 p=2.3e−22
Mean:0.03 Mean:0.04 p=0.502
Mean:0 Mean:0 p=0.582
Mean:0.07 Mean:0.05 p=0.128
Mean:0.2 Mean:0.32 p=4.35e−12
Mean:0.03 Mean:0.04 p=0.002
Mean:0 Mean:0 p=0.791
Mean:0.01 Mean:0.02 p=7.9e−05
Mean:0.13 Mean:0.19 p=4.2e−09
Mean:0.06 Mean:0.05 p=0.078
Mean:0.04 Mean:0.03 p=0.012
Mean:0.03 Mean:0.02 p=0.003
Mean:0.5 Mean:0.48 p=0.471

**Supplementary figS2**. **A-C.** Cell state plot of the neoplastic cells negative for γ-H2AX immunofluorescence (DNA Damage negative- top) and positive for γ-H2AX immunofluorescence (DNA Damage positive-bottom) in (A) GBM explant, (B) IRGBM1, or (C) IRGBM2. **D.** Proportion of stem (GSC- Glioma stem-like cells, CSC- Cancer stem-like cells) and non-stem like neoplastic cells in brain tumor tissues. **E.** Cellular composition of the brain tumor tissues showing that tumor associated macrophages (TAMs) are the most abundant non-malignant cell type populating all the tissues studied. **F-I.** Comparison of cell counts of brain microenvironment specific non-malignant cells other than microglia in fast repair and slow repair zones in GBM explant (F), LungMet (G), IRGBM1 (H), IRGBM2 (I). P values calculated using Wilcoxon test.

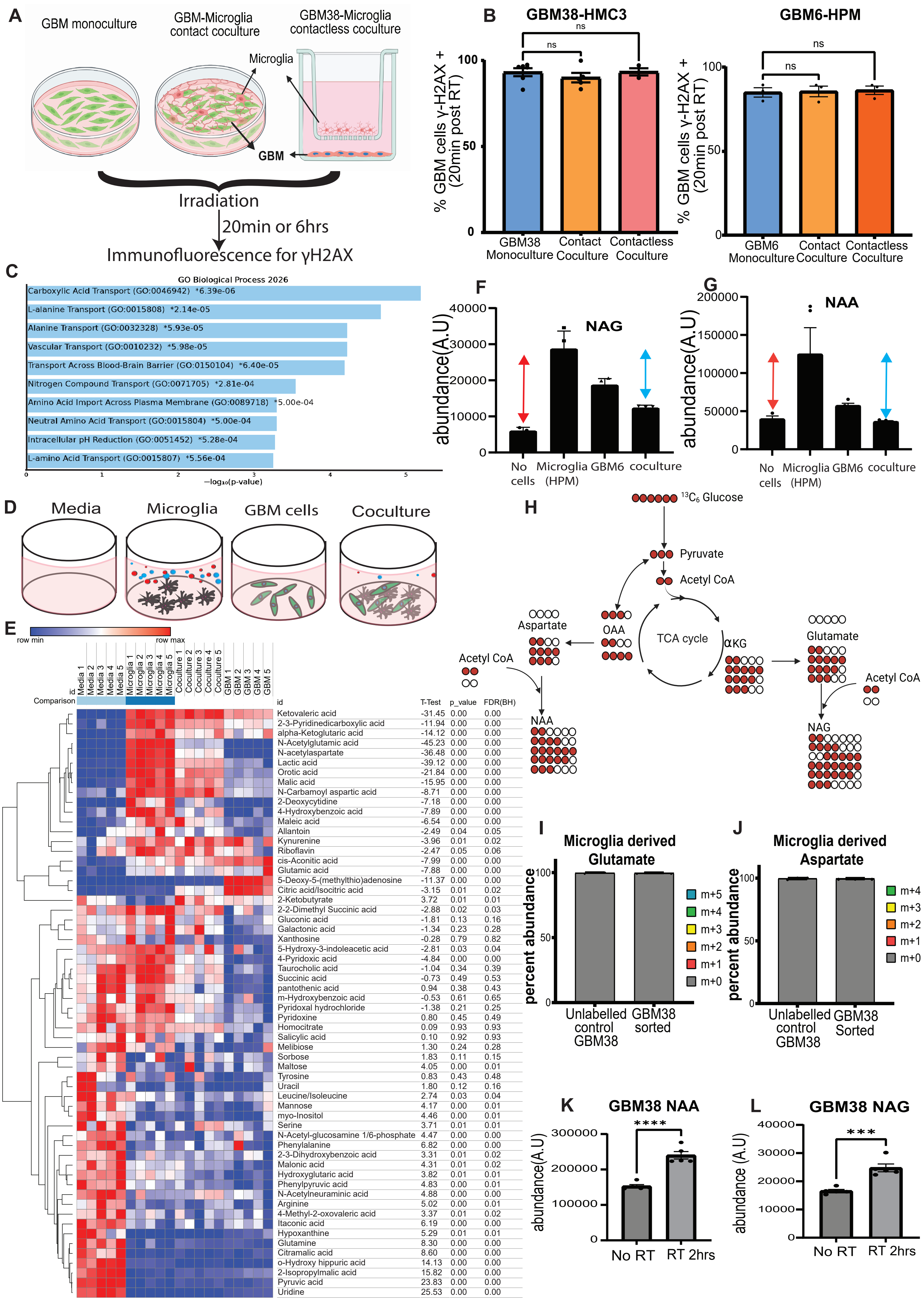


| id | T-Test | p_value | FDR(BH) |
|---|---|---|---|
| Ketovaleric acid | -31.45 | 0.00 | 0.00 |
| 2-3-Pyridinedicarboxylic acid | -11.94 | 0.00 | 0.00 |
| alpha-Ketoglutaric acid | -14.12 | 0.00 | 0.00 |
| N-Acetylglutamic acid | -45.23 | 0.00 | 0.00 |
| N-acetylaspartate | -36.48 | 0.00 | 0.00 |
| Lactic acid | -39.12 | 0.00 | 0.00 |
| Orotic acid | -21.84 | 0.00 | 0.00 |
| Malic acid | -15.95 | 0.00 | 0.00 |
| N-Carbamoyl aspartic acid | -8.71 | 0.00 | 0.00 |
| 2-Deoxycytidine | -7.18 | 0.00 | 0.00 |
| 4-Hydroxybenzoic acid | -7.89 | 0.00 | 0.00 |
| Maleic acid | -6.54 | 0.00 | 0.00 |
| Allantoin | -2.49 | 0.04 | 0.05 |
| Kynurenine | -3.96 | 0.01 | 0.02 |
| Riboflavin | -2.47 | 0.05 | 0.06 |
| cis-Aconitic acid | -7.99 | 0.00 | 0.00 |
| Glutamic acid | -7.88 | 0.00 | 0.00 |
| 5-Deoxy-5-(methylthio)adenosine | -11.37 | 0.00 | 0.00 |
| Citric acid/Isocitric acid | -3.15 | 0.01 | 0.02 |
| 2-Ketobutyrate | 3.72 | 0.01 | 0.01 |
| 2-2-Dimethyl Succinic acid | -2.88 | 0.02 | 0.03 |
| Gluconic acid | -1.81 | 0.13 | 0.16 |
| Galactonic acid | -1.34 | 0.23 | 0.28 |
| Xanthosine | -0.28 | 0.79 | 0.82 |
| 5-Hydroxy-3-indoleacetic acid | -2.81 | 0.03 | 0.04 |
| 4-Pyridoxic acid | -4.84 | 0.00 | 0.00 |
| Taurocholic acid | -1.04 | 0.34 | 0.39 |
| Succinic acid | -0.73 | 0.49 | 0.53 |
| pantothenic acid | 0.94 | 0.38 | 0.43 |
| m-Hydroxybenzoic acid | -0.53 | 0.61 | 0.65 |
| Pyridoxal hydrochloride | -1.38 | 0.21 | 0.25 |
| Pyridoxine | 0.80 | 0.45 | 0.49 |
| Homocitrate | 0.09 | 0.93 | 0.93 |
| Salicylic acid | 0.10 | 0.92 | 0.93 |
| Melibiose | 1.30 | 0.24 | 0.28 |
| Sorbose | 1.83 | 0.11 | 0.15 |
| Maltose | 4.05 | 0.00 | 0.01 |
| Tyrosine | 0.83 | 0.43 | 0.48 |
| Uracil | 1.80 | 0.12 | 0.16 |
| Leucine/Isoleucine | 2.74 | 0.03 | 0.04 |
| Mannose | 4.17 | 0.00 | 0.01 |
| myo-Inositol | 4.46 | 0.00 | 0.01 |
| Serine | 3.71 | 0.01 | 0.01 |
| N-Acetyl-glucosamine 1/6-phosphate | 4.47 | 0.00 | 0.01 |
| Phenylalanine | 6.82 | 0.00 | 0.00 |
| 2-3-Dihydroxybenzoic acid | 3.31 | 0.01 | 0.02 |
| Malonic acid | 4.31 | 0.00 | 0.01 |
| Hydroxyglutaric acid | 3.82 | 0.01 | 0.01 |
| Phenylpyruvic acid | 4.83 | 0.00 | 0.01 |
| N-Acetylneuraminic acid | 4.88 | 0.00 | 0.00 |
| Arginine | 5.02 | 0.00 | 0.01 |
| 4-Methyl-2-oxovaleric acid | 3.37 | 0.01 | 0.02 |
| Itaconic acid | 6.19 | 0.00 | 0.00 |
| Hypoxanthine | 5.29 | 0.00 | 0.01 |
| Glutamine | 8.30 | 0.00 | 0.00 |
| Citramalic acid | 8.60 | 0.00 | 0.00 |
| o-Hydroxy hippuric acid | 14.13 | 0.00 | 0.00 |
| 2-Isopropylmalic acid | 15.82 | 0.00 | 0.00 |
| Pyruvic acid | 23.83 | 0.00 | 0.00 |
| Uridine | 25.53 | 0.00 | 0.00 |

**Supplementary figS3. A.** Schematic depicting monoculture and coculture systems employed to study DNA repair phenotype in GBM cells. **B.** Quantification of γ-H2AX$^+$ GBM cells 20 minutes after RT (4 Gy) in monoculture and coculture systems, as indicated. P values calculated using one-way Anova. (n=5 [obtained from 2 experiments] for GBM38 monoculture, n=6 [2 experiments, 3 replicates each] for GBM38-HMC3 contact cocultures and n=3 for GBM38-HMC3 contactless cocultures; n=3 for each condition in GBM6-HPM coculture experiment). **C.** Gene ontology of commonly upregulated transporters in myeloid cells present in fast repair zones in the irradiated human brain tumor cohort described in Fig 2. **D.** Illustration of experimental design of conditioned media metabolomic analysis. **E.** Heatmap showing N-acetyl glutamate (NAG) and N-acetyl aspartate (NAA) as the metabolites absent or low in the control media and GBM38 conditioned media, high in microglia (HMC3) conditioned media and reduced in coculture conditioned media. T test shows comparison between control media and microglia (HMC3) conditioned media. **F.** Abundance of N-acetyl glutamate (NAG) in conditioned media of monocultures and coculture of GBM6 and HPM microglia cells showing secretion by microglia(red arrow) and reduced abundance in cocultures(blue arrow, n=4 biologic replicates per condition). **G.** Abundance of N-acetyl aspartate (NAA) in conditioned media of monocultures and coculture of GBM6 and HPM microglia cells showing secretion by microglia (red arrow) and reduced levels in cocultures (blue arrow, n=4 biologic replicates). **H.** Schematic illustration of isotope tracing with uniformly labeled Glucose carbons ($^{13}C_6$ glucose). Each red dot represents a heavy ($^{13}C$) carbon. **I.** Percent abundance of microglia derived $^{13}C$ labelled glutamate in GBM38 cells (n=3 unlabelled controls, n=5 replicates of GBM cells sorted from coculture with microglia precultured with $^{13}C_6$ glucose). **J**. Percent abundance of microglia derived $^{13}C$ labelled aspartate in GBM38 cells (n=3 unlabelled controls, n=5 replicates of GBM cells sorted from coculture with microglia precultured with $^{13}C_6$ glucose). **K.** Intracellular abundance of NAG in GBM38 cells 2 hrs after 8 Gy RT. P value calculated using unpaired T test. **L.** Intracellular abundance of NAA in GBM38 cells 2 hrs after 8 Gy radiation. P value calculated using unpaired T test. P values indicated as * 0.01 to 0.05, ** 0.001 to 0.01, *** 0.0001 to 0.001, ****<0.0001. All error bars indicate SEM.

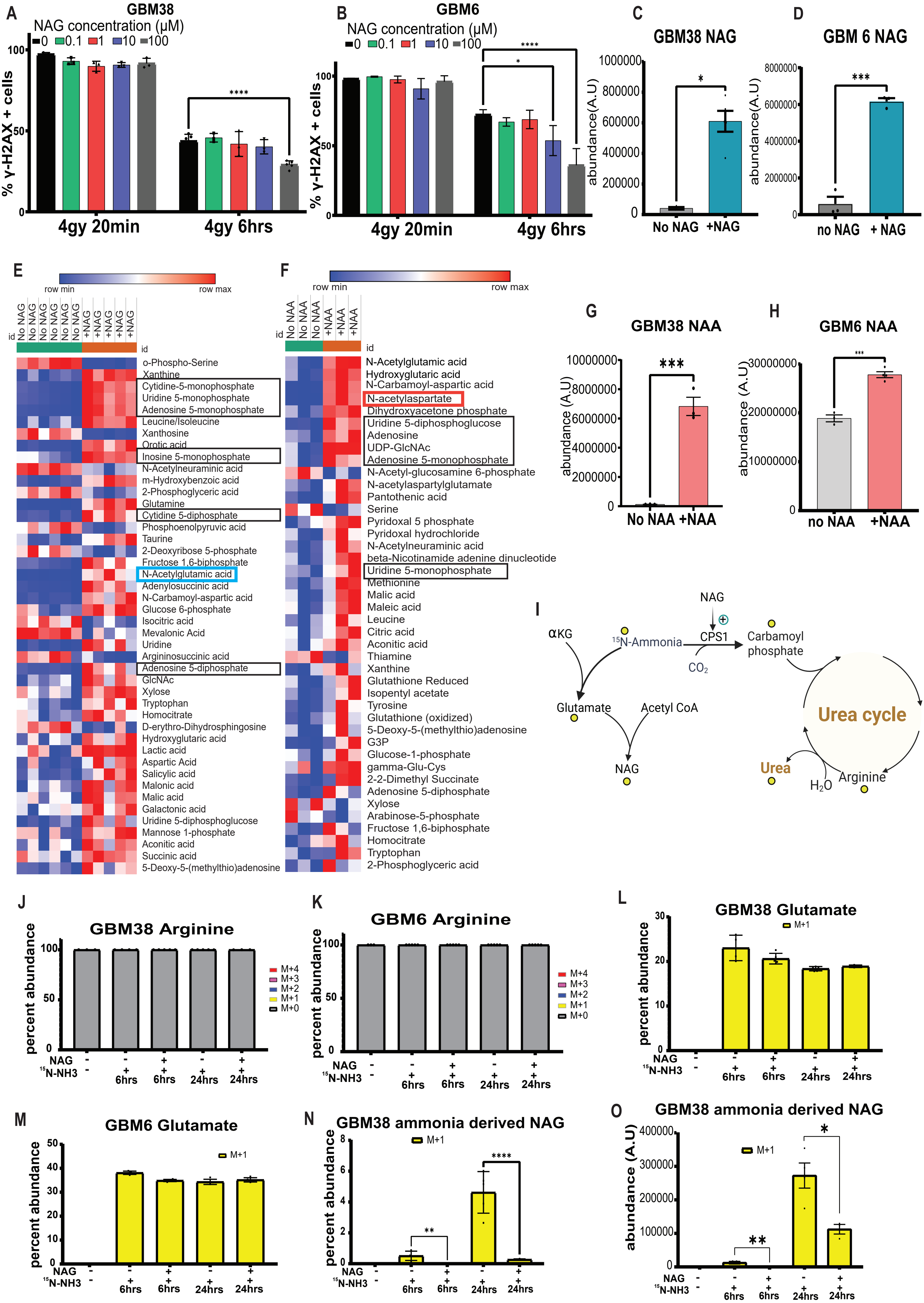

A
GBM38
NAG concentration (μM)
0 0.1 1 10 100
% γ-H2AX + cells
4gy 20min
4gy 6hrs
****
B
GBM6
NAG concentration (μM)
0 0.1 1 10 100
% γ-H2AX + cells
4gy 20min
4gy 6hrs
****
*
C
GBM38 NAG
abundance(A.U)
No NAG
+NAG
*
D
GBM 6 NAG
abundance(A.U)
no NAG
+ NAG
***
E
row min
row max
id
No NAG
+NAG
o-Phospho-Serine
Xanthine
Cytidine-5-monophosphate
Uridine 5-monophosphate
Adenosine 5-monophosphate
Leucine/Isoleucine
Xanthosine
Orotic acid
Inosine 5-monophosphate
N-Acetylneuraminic acid
m-Hydroxybenzoic acid
2-Phosphoglyceric acid
Glutamine
Cytidine 5-diphosphate
Phosphoenolpyruvic acid
Taurine
2-Deoxyribose 5-phosphate
Fructose 1,6-biphosphate
N-Acetylglutamic acid
Adenylosuccinic acid
N-Carbamoyl-aspartic acid
Glucose 6-phosphate
Isocitric acid
Mevalonic Acid
Uridine
Argininosuccinic acid
Adenosine 5-diphosphate
GlcNAc
Xylose
Tryptophan
Homocitrate
D-erythro-Dihydrosphingosine
Hydroxyglutaric acid
Lactic acid
Aspartic Acid
Salicylic acid
Malonic acid
Malic acid
Galactonic acid
Uridine 5-diphosphoglucose
Mannose 1-phosphate
Aconitic acid
Succinic acid
5-Deoxy-5-(methylthio)adenosine
F
row min
row max
id
No NAA
+NAA
N-Acetylglutamic acid
Hydroxyglutaric acid
N-Carbamoyl-aspartic acid
N-acetylaspartate
Dihydroxyacetone phosphate
Uridine 5-diphosphoglucose
Adenosine
UDP-GlcNAc
Adenosine 5-monophosphate
N-Acetyl-glucosamine 6-phosphate
N-acetylaspartylglutamate
Pantothenic acid
Serine
Pyridoxal 5 phosphate
Pyridoxal hydrochloride
N-Acetylneuraminic acid
beta-Nicotinamide adenine dinucleotide
Uridine 5-monophosphate
Methionine
Malic acid
Maleic acid
Leucine
Citric acid
Aconitic acid
Thiamine
Xanthine
Glutathione Reduced
Isopentyl acetate
Tyrosine
Glutathione (oxidized)
5-Deoxy-5-(methylthio)adenosine
G3P
Glucose-1-phosphate
gamma-Glu-Cys
2-2-Dimethyl Succinate
Adenosine 5-diphosphate
Xylose
Arabinose-5-phosphate
Fructose 1,6-biphosphate
Homocitrate
Tryptophan
2-Phosphoglyceric acid
G
GBM38 NAA
abundance (A.U)
No NAA
+NAA
***
H
GBM6 NAA
abundance (A.U)
no NAA
+NAA
***
I
NAG
αKG
15N-Ammonia
CPS1
CO2
Carbamoyl phosphate
Glutamate
Acetyl CoA
NAG
Urea cycle
Urea
H2O
Arginine
J
GBM38 Arginine
percent abundance
M+4 M+3 M+2 M+1 M+0
NAG
15N-NH3
6hrs 6hrs 24hrs 24hrs
K
GBM6 Arginine
percent abundance
M+4 M+3 M+2 M+1 M+0
NAG
15N-NH3
6hrs 6hrs 24hrs 24hrs
L
GBM38 Glutamate
M+1
percent abundance
NAG
15N-NH3
6hrs 6hrs 24hrs 24hrs
M
GBM6 Glutamate
M+1
percent abundance
NAG
15N-NH3
6hrs 6hrs 24hrs 24hrs
N
GBM38 ammonia derived NAG
M+1
percent abundance
**
****
NAG
15N-NH3
6hrs 6hrs 24hrs 24hrs
O
GBM38 ammonia derived NAG
M+1
abundance (A.U)
**
*
NAG
15N-NH3
6hrs 6hrs 24hrs 24hrs

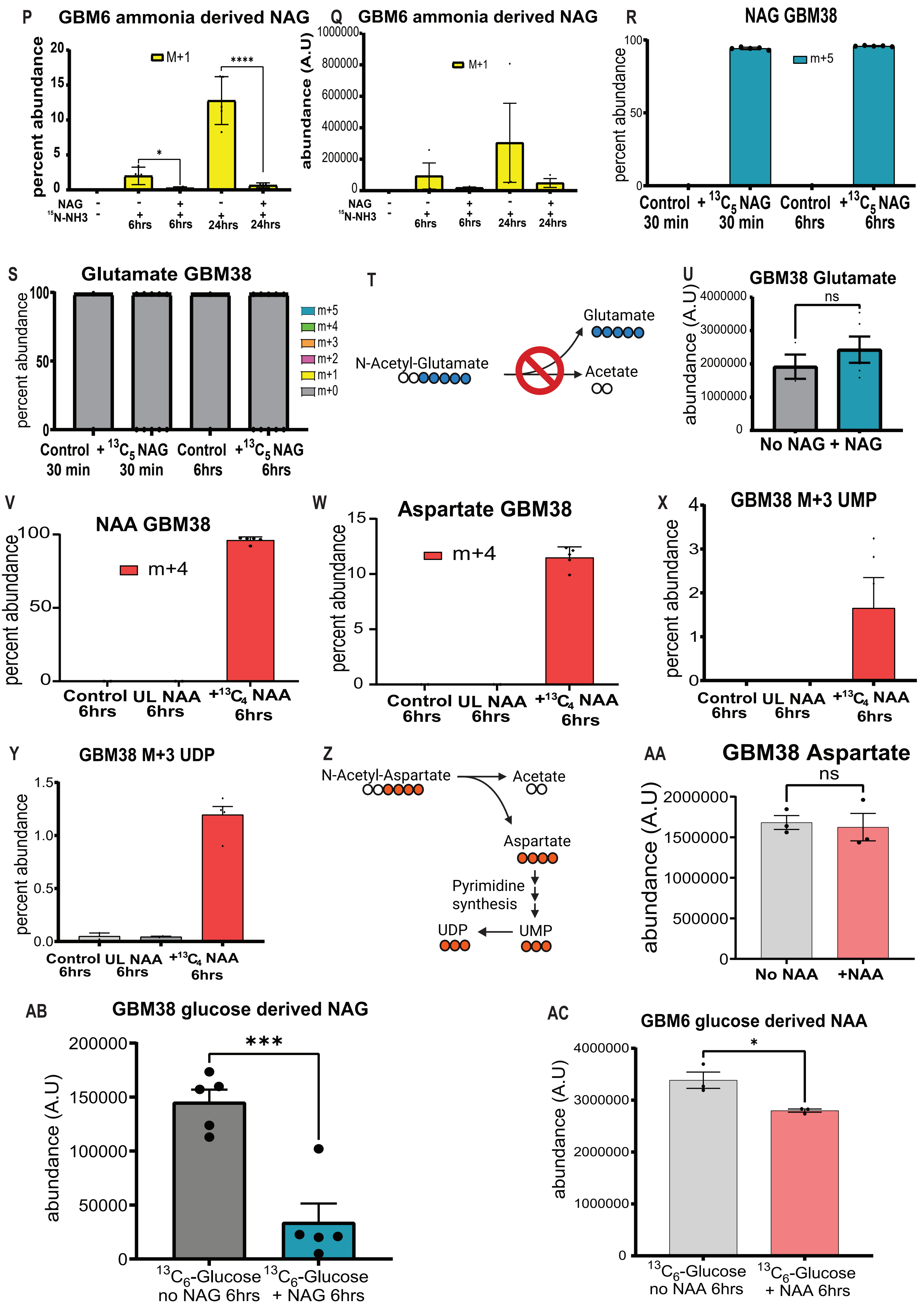

P
GBM6 ammonia derived NAG
percent abundance
M+1
NAG
15N-NH3
6hrs
24hrs
Q
GBM6 ammonia derived NAG
abundance (A.U)
M+1
R
NAG GBM38
percent abundance
m+5
Control 30 min
+ 13C5 NAG 30 min
Control 6hrs
+13C5 NAG 6hrs
S
Glutamate GBM38
percent abundance
m+5
m+4
m+3
m+2
m+1
m+0
T
N-Acetyl-Glutamate
Glutamate
Acetate
U
GBM38 Glutamate
abundance (A.U)
ns
No NAG
+ NAG
V
NAA GBM38
percent abundance
m+4
Control 6hrs
UL NAA 6hrs
+13C4 NAA 6hrs
W
Aspartate GBM38
percent abundance
m+4
X
GBM38 M+3 UMP
percent abundance
Y
GBM38 M+3 UDP
percent abundance
Z
N-Acetyl-Aspartate
Acetate
Aspartate
Pyrimidine synthesis
UMP
UDP
AA
GBM38 Aspartate
abundance (A.U)
ns
No NAA
+NAA
AB
GBM38 glucose derived NAG
abundance (A.U)
13C6-Glucose no NAG 6hrs
13C6-Glucose + NAG 6hrs
AC
GBM6 glucose derived NAA
abundance (A.U)
13C6-Glucose no NAA 6hrs
13C6-Glucose + NAA 6hrs

**Supplementary fig4. A, B.** Concentration-response graph of effect of NAG supplementation on DNA repair in GBM38 cells(A; n=3 biologic replicates in all conditions except RT 6hrs no NAG and 100uM NAG which have 5 biologic replicates each from 2 experiments) and GBM6 cells(B; n=3 biologic replicates in all conditions). **C,D.** Abundance of intracellular NAG in GBM 38 (C; n=3 biologic replicates in no NAG control and n=5 biologic replicates in NAG supplemented condition) and GBM6 (D; n= 6 biologic replicates for no NAG control, n=5 for NAG supplemented condition) cells. Pvalues calculated using unpaired T-test. E. Heatmap showing intracellular abundance of metabolites that are significantly different in GBM6 control cells and GBM6 cells supplemented with NAG. F. Heatmap showing intracellular abundance of metabolites that are significantly different in GBM38 control cells and GBM38 cells supplemented with NAA. G,H. Quantification of abundance of intracellular NAA in GBM 38 (G; n=3 biologic replicates for each condition) and GBM6 (H; n=4 biologic replicates for each condition) cells. P value calculated using unpaired T- test. I. Schematic illustrating stable isotope tracing with 15N ammonia. The yellow dot represents the heavy (15N) nitrogen.J,K. Graphs showing percent abundance of 15N ammonia-derived arginine in GBM38(J) cells and GBM6 (K) with or without exogenous NAG supplementation. L,M. Percent abundance of 15N ammonia-derived glutamate in GBM38 (L) and GBM6 (M) cells with or without exogenous NAG supplementation. N. Percent abundance of 15N ammonia-derived NAG in GBM38 cells with or without exogenous NAG supplementation. O. Total abundance of 15N ammonia-derived NAG in GBM38 cells with or without exogenous NAG supplementation. P. Percent abundance of 15N ammonia-derived NAG in GBM6 cells with or without exogenous NAG supplementation. Q. Total abundance of 15N ammonia-derived NAG in GBM38 cells with or without exogenous NAG supplementation.(For all 15N ammonia tracing experiments in GBM38, n=3 for control condition, n=4 for no NAG +15N ammonia, n=5 for 15N ammonia +NAG 6hrs, n=5 for no NAG+ 15N ammonia 24hrs and n=3 for 15N ammonia + NAG condition; For GBM6, n=5 for all conditions) R,S. 13C5 NAG tracing in GBM38 cells. Graph showing percent abundance of intracellular m+5 tracer-derived NAG 30 min and 6hrs after treatment(R). Graph depicting percent abundance of 13C5 NAG derived glutamate, showing near complete endogenous (m+0) glutamate in GBM38 cells(S). T. Schematic illustrating the summary of observations in R and S. U. Total abundance of Glutamate in GBM38 cells treated with NAG for 6 hrs (n=3 biologic replicates in no NAG control and n=5 biologic replicates in NAG supplemented condition). V-Y. 13C4 NAA tracing in GBM38 cells 6hrs after supplementation of tracer (n=2 biologic replicates for control condition, n=2 biological replicates for unlabeled NAA control condition [UL NAA] and n=5 biologic replicates for the tracer supplemented cells [13C4 NAA]). Percent abundance of intracellular m +4 tracer derived NAA in GBM38 cells (V). Percent abundance of 13C4 NAA derived Aspartate (m+4) in GBM38 cells(W). Percent abundance of tracer derived UMP (m+3) in GBM38 cells(X). Percent abundance of tracer derived UDP (m+3) in GBM38 cells(Y). Z. Schematic illustrating the summary of observations in V-Y. AA. Total abundance of aspartate in GBM38 cells treated with NAA for 6 hrs(n=3 biological replicates for each condition). AB. Total abundance of glucose-derived NAG in GBM 38 cells cultured with 13C6 glucose for 6 hrs with or without exogenous supplementation of NAG(n=5 biologic replicates for each condition). AC. Total abundance of glucose-derived NAA in GBM6 cells cultured with 13C6 glucose for 6 hrs with or without exogenous supplementation of NAA(n=3 biologic replicates for each condition). All p- values were calculated using unpaired T-test and indicated as * 0.01 to 0.05, ** 0.001 to 0.01, *** 0.0001 to 0.001, ****<0.0001, Error bars show SEM.

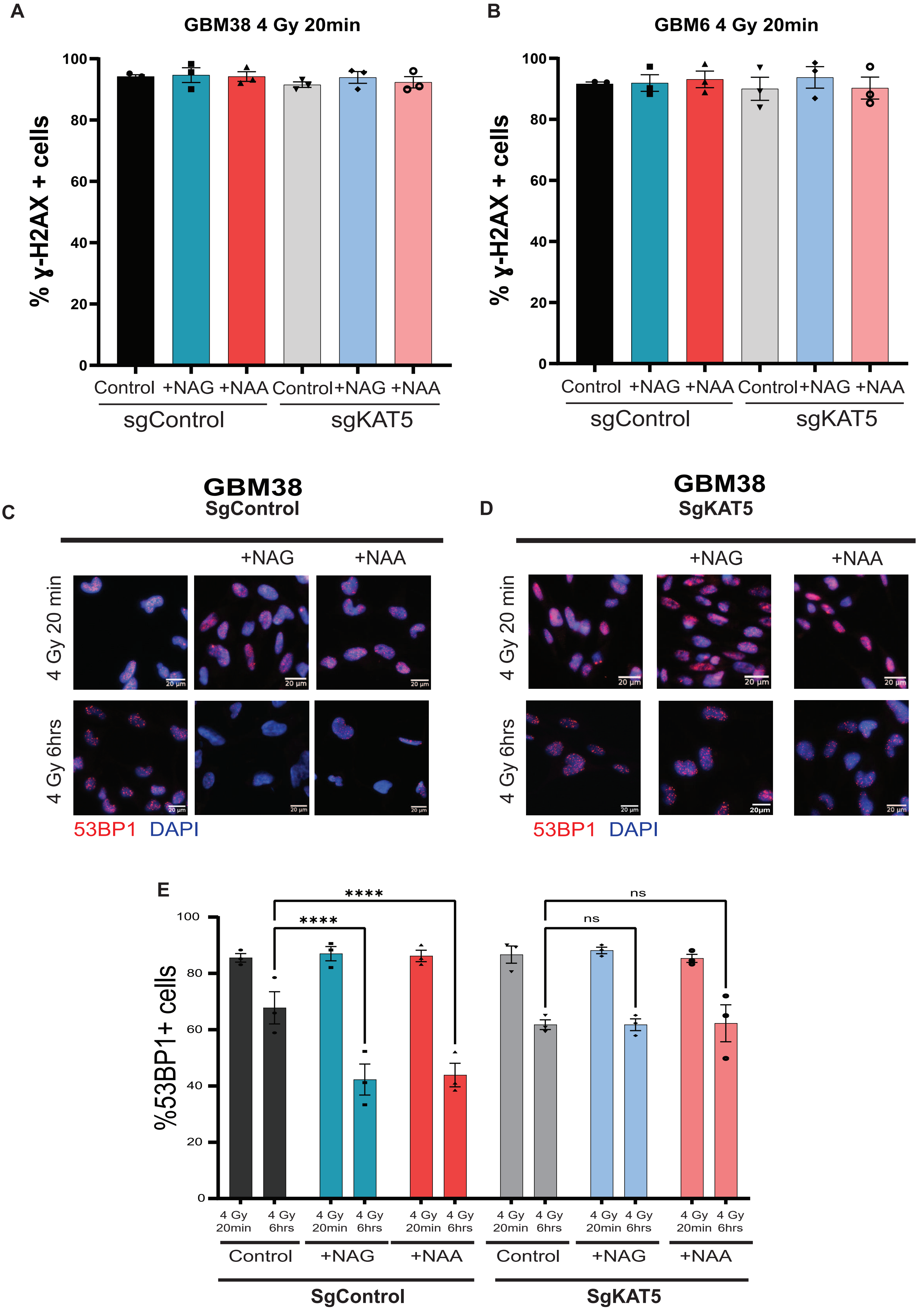
A
GBM38 4 Gy 20min
% γ-H2AX + cells
Control +NAG +NAA Control +NAG +NAA
sgControl
sgKAT5
B
GBM6 4 Gy 20min
% γ-H2AX + cells
Control +NAG +NAA Control +NAG +NAA
sgControl
sgKAT5
C
GBM38
SgControl
+NAG
+NAA
4 Gy 20 min
4 Gy 6hrs
20 µm
53BP1 DAPI
D
GBM38
SgKAT5
+NAG
+NAA
4 Gy 20 min
4 Gy 6hrs
20 µm
53BP1 DAPI
E
%53BP1+ cells
****
****
ns
ns
4 Gy 20min
4 Gy 6hrs
Control
+NAG
+NAA
SgControl
SgKAT5

**Supplementary figS5 (supporting Fig 6).A,B.** Quantification of γ-H2AX positive cells in sgControl and sgKAT5 GBM38 cells (A) and GBM6 cells (B) 20min after RT (4 Gy). **C,D**. Representative images of 53BP1 immunofluorescence in GBM38 CRISPR control (sgControl) cells (C) and KAT5 Knockdown (sgKAT5) GBM38 cells (D) supplemented with NAG or NAA 20 min and 6hrs after RT (4 Gy). **C.** Quantification of 53BP1 positivity of sgControl and sgKAT5 GBM38 cells supplemented with NAG or NAA 6hrs after exposure to 4 Gy RT. (n=3 biologic replicates for each condition). P values calculated using unpaired T tests and indicated as * 0.01 to 0.05, ** 0.001 to 0.01, *** 0.0001 to 0.001, ****<0.0001. Error bars indicate SEM.

**Supplementary Methods:**

**Patient tissue samples for spatial transcriptomics:**

Human GBM and metastatic lung cancer tissue for ST on visium HD platform(Fig 2A):

1. Irradiated explant of human GBM tissue (GBM explant)- Tumor tissue sample was obtained from a female patient aged 51yrs, undergoing surgical resection as a part of standard of care for GBM. The tissue was cut into approximately 6 mm x 6 mm chunks of varying thickness and cultured ex vivo in serum free media (complete neurosphere media described below). The tissue explant used for ST was irradiated with 12 Gy single dose X-ray irradiation shortly after suspension in serum free media and cultured for 6 hrs after exposure to radiation. At the end of 6hrs, the explant was fixed in 10% formaldehyde and embedded in paraffin (FFPE). This FFPE tissue was sectioned at 4 μm thickness. Glioblastoma WHO grade 4, IDH1 wild type (IHC), MGMT promoter methylation equivocal.
2. Irradiated patient GBM 1(IRGBM1)- Brain tumor tissue obtained from surgical resection 1 day post irradiation with fractionated Stereotactic Body Radiotherapy (SBRT) (27 Gy in 3 fractions) from a female patient aged 48yrs with a history of extracranial breast cancer and later diagnosed to be GBM was analyzed for this study. Diagnosis: GLIOBLASTOMA, IDH-WILDTYPE, CNS WHO grade 4, MGMT promoter methylation: Negative.
3. Irradiated patient GBM 2 (IRGBM2)- Brain tumor tissue obtained from surgical resection 3days post irradiation with a single dose Stereotactic radiosurgery (SRS) with 18 Gy to the 50% isodose line from a male patient aged 78years, with a history of extracranial lung cancer and later diagnosed to be GBM was analyzed for this study. Diagnosis: GLIOBLASTOMA, IDH-WILDTYPE, CNS WHO grade 4, MGMT promoter methylation positive.
4. Lung mets- Brain tumor tissue obtained from surgical resection 1 day post irradiation with a single dose Stereotactic radiosurgery (SRS) with 18Gy of a metastatic non-small cell lung cancer to the brain from a male patient aged 44 yrs at the University of Michigan was included in this study. Diagnosis: Poorly differentiated lung adenocarcinoma with a RET fusion (KIF5B-RET).

**Spatial transcriptomics:**

**Visium:** Processing and Quality Control of Visium ST Data

Spots with fewer than 200 expressed genes were labeled as low quality and excluded from downstream analyses. Similarly, spots with over 5% hemoglobin genes were excluded due to excessive bleeding in tissue. Any slide with fewer than 100 spots passing quality-control (QC) thresholds was not used for integrative analysis. Slides passing QC for each cohort were independently merged into a single *Seurat* object. Count data analysis was carried out using

*Seurat v5.2* [1]. Raw counts were normalized using the *NormalizeData* function. The top 3000 variable genes were used for dimension reduction using PCA with the *RunPCA* function, and the top 15 PCs were used to construct a UMAP data embedding using *RunUMAP* for sample visualization in the UM cohort.

**Visium Cytassist HD**:

Quantification of DNA damage and signal filtering

Following the generation of custom segmented spatial matrices, DNA damage levels were quantified by extracting immunofluorescence signal intensities for the $\gamma$H2AX (Red) and DAPI (Blue) channels within each segmented nuclear boundary. To account for varying signal to noise ratios and background fluorescence across the four cohorts, specialized thresholding strategies were implemented for each sample:

- For the **LungMets** sample, a fixed mean intensity threshold of $\mathbf{25}$ was applied.
- For the **GBM explant**, a high stringency dynamic threshold of $\mathbf{0.364}$ (calculated as **2x the median** mean intensity) was utilized to selectively capture only the most severely damaged nuclei within the *ex vivo* irradiated environment.
- **Irradiated patient GBM1 and Irradiated patient GBM2:** To address staining variability in patient samples, ratio-based normalization was employed.
    - For **IRGBM1**, a strictly ratio-based logic was implemented, classifying cells as "Damage-High" if the ratio of Red to Blue intensity ($Ratio = \frac{\bar{I}_{red}}{\bar{I}_{blue}}$) exceeded $\mathbf{0.25}$.
    - For **IRGBM2**, an **enhanced hybrid logic** was used to account for higher background levels: cells were classified as "Damage-High" if the mean Red intensity exceeded $\mathbf{28}$ or the R/B ratio exceeded $\mathbf{0.18}$.

These classifications were integrated into the **Seurat (v5.4.0)** [1] metadata as a binary "DNA damage status" (Damage-High vs Damage-Low) for downstream spatial analysis.

Malignant state and lineage hierarchy analysis

To characterize the phenotypic diversity of neoplastic cells, relative lineage and differentiation scores were calculated based on established glioblastoma cellular states (AC, MES, NPC, OPC). For each custom segmented cell, prediction scores for these four states were extracted from the reference based annotation. Quadrant coordinates were calculated by determining the maximum score between the bottom lineages (AC vs MES) and top progenitor states (NPC vs OPC). A relative lineage score ($x$) and a relative differentiation score ($y$) were derived using a log scaled ratio of these intensities:

$$x = sign(x_{raw}) \cdot log_2(|x_{raw}| + 1)$$

$$y = sign(y_{raw}) \cdot log_2(|y_{raw}| + 1)$$

These coordinates were used to project neoplastic cells into a 2D hierarchy, often referred to as a "butterfly" plot to visualize state shifts associated with DNA damage [2].

**Stemness scoring and classification of neoplastic cells**

To quantify stemness features within neoplastic tissue domains, analysis was restricted strictly to cells annotated as Neoplastic. A composite stemness score was calculated for each individual cell using Ucell score based on a panel of canonical stem cell markers: PROM1 (CD133), SOX2, OLIG2, and NES (Nestin). Within each sample, neoplastic cells were analyzed using dimension reduction and construction of UMAPs for identifying subpopulations. The clusters with the highest GSC score were annotated as Stem-like cells (Glioma stem cells: GSC or Cancer TSem Cells (CSC) The remaining clusters were designated as non-stem-like (nonGSC or nonCSC). These stemness states were subsequently integrated with spatial DNA damage annotations (Damage_Low vs. Damage_High) to quantify the proportion and distribution of stem-like states across DNA damage microenvironments.

<u>Computational infrastructure and data integration</u>

Large scale image processing and **Space Ranger HD** workflows were conducted on the **University of Michigan Great Lakes** high performance computing cluster [3]. Analysis utilized high-memory nodes (up to 180 $GB$ RAM) and NVIDIA GPUs for Cellpose-accelerated segmentation. Spatial coordinates and cell level metadata including DNA damage status and transcriptomic profiles were integrated for downstream analysis using **Seurat (v5.4.0)** [1]. This infrastructure ensured a spatially resolved, single cell level assessment of the tumor microenvironment across all experimental and clinical cohorts.

**Multiplex Immunofluorescence:**

FFPE sections from Irradiated brain TRP tumor and irradiated GBM explant tissues described above were used to study TAM abundance in fast DNA repair regions using an orthogonal method to detect the cell type based on protein expression for IBA1 in regions with low DNA damage, detected using $\gamma$H2AX .

Multiplex IF staining was performed using the Opal protocol, as described previously[4]. Briefly, the staining protocol for each individual primary antibody was first optimized and each of the sections mentioned above were sequentially stained with a panel of antibodies (Supplementary methods, Table1) using a Ventana Discovery Ultra stainer (Ventana Medical Systems). After incubation with the primary antibody followed by a Tris-buffered saline with Tween-20 buffer (TBST) wash, Ventana OmniMap anti-rabbit horseradish peroxidase followed by Opal Tyramide

Signal Amplification were applied. This creates a covalent bond between the tissue and the fluorophore at the horseradish peroxidase site. Following this, heat-induced antigen retrieval was performed using CC2 buffer to remove the primary antibody/secondary antibody complex, and the next antibody was applied. A panel of 6 fluorochromes was sequentially applied to each tissue section. The slides were then mounted with ProLong Gold containing DAPI and scanned using an Akoya Polaris IF scanner at 20× magnification. The images were visualized on Phenochart to perform spectral unmixing and exported to qupath for neighborhood analysis.

| Primary Antibody | Host Species | Clone | Company | Catalogue No. | Antibody Dilution | Fluorochrome | Fluorochrome Dilution | Staining Position |
|---|---|---|---|---|---|---|---|---|
| pS139 Histone H2a.x | Rabbit | 20E3 | CST | 9718 | 1:100 | Opal 620 | 1:150 | 4 |
| IBA1 | Rabbit | E404W | CST | 17198 | 1:1000 | Opal 480 | 1:150 | 5 |

Table1. Antibody panel used for multiplex IF.

**IF for γ-H2AX and 53BP1:**

GBM cells and microglia were cultured as adherent cultures on coverslips as described in the methods and irradiated at 4 Gy. The cells were fixed using 4% Paraformaldehyde (PFA, Thermo Scientific, Catalog No.: J61899-AK) in PBS for 20 minutes at room temperature, at 3 different time points post-irradiation: 20 minutes, 2 hours, and 6 hours. The fixed cells were stored at 4 degrees until further processing. The cells fixed to the coverslips were permeabilized by treating with 0.2% Triton X-100 solution (Triton X-100 [Alfa Aesar, Catalog No. A16046-AE]) for 15 minutes at room temperature. Following permeabilization, the cells were treated with IF blocking buffer for 1 hour at room temperature and then incubated overnight at 4°C with a the appropriate primary antibody (γ-H2AX :P-Histone H2AX S139 20E3 rabbit mAb, 99µg/mL, Cell Signaling, Catalog No. 9718, 1:1000 dilution; 53BP1-Cell Signaling, 4937S, 1:100 dilution). The cells were then incubated with Alexa Fluor 594 secondary antibody (Alexa Fluor 594 -goat pAb to rabbit IgG, 2mg/mL, Abcam ab150080) and counterstained with DAPI (Sigma, Catalog No. D9542).

FFPE tissues obtained from TRP flank and brain tumors obtained as described above were sectioned at 4um thickness onto charged glass slides. These sections were backed at 56$^{o}$C for 2hrs, treated with Xylene to deparaffinize the sections followed by gradient ethanol (100%, 95% and 70%) and water. Antigen retrieval was performed using Retrieve-All Antigen Unmasking solution (Biolegend, 927901). The sections were then treated with IF blocking buffer and incubated with the appropriate primary antibody (γ-H2AX :P-Histone H2AX S139 20E3 rabbit mAb, 99µg/mL, Cell Signaling, Catalog No. 9718, 1:1000 dilution; 53BP1-Cell Signaling, 4937S, 1:100 dilution)

**Fluorescence Microscopy and Analysis:**

Fluorescent images were captured using the BioTek Cytation 5 confocal fluorescence microscope. For each replicate, three representative fields were imaged and analyzed using Gen5 software (version 3.12).

Images were processed using cell segmentation to identify individual cells and nuclei with the use of DAPI for primary masking. γ-H2AX or 53BP1 foci within each nucleus were detected using secondary masks based on Texas Red fluorescence (586–647 nm). Along with counting the number of foci, the average Texas Red signal intensity was also measured and used to define γ-H2AX "positive" cells which represented significant DNA damage. All conditions being compared were imaged using fixed exposure settings for the different channels.

Cells were classified as γ-H2AX positive based on both the number of foci (≥10 foci per nucleus) and the overall Texas Red fluorescence intensity (Thresholds were determined based on positive and negative controls in each experiment). Cells that did not meet these thresholds were considered "negative". This was used as a measure of tracking DNA damage repair at the various time points following radiation treatment.

Microsoft Excel (Office 365, v2402) was used to compile and standardize the data. For each coverslip, the percentage of γ-H2AX positive cells in 3 field and averaged across three different fields to include a minimum of 100 cells per biological replicate). A minimum of 3 separate coverslips were imaged to obtain a minimum of 3 biological replicates for each experiment.

Statistical comparisons between each of the treatment and time point conditions were performed using an unpaired t-test in GraphPad Prism (version 10.4.1), and results were considered significant at $P < 0.05$.

For the tissue sections, the cells containing at least 10 foci per nucleus and higher than the threshold intensity for Texas red were defined as positive for 53BP1 or γ-H2AX. 3 separate fields within each tissue were assessed for 53BP1 foci and γ-H2AX positive cells and 3 separate tissues were analyzed for this study.

**Cell Culture:**

Human GBM cell lines: GBM38 and GBM6 cell lines

GBM38 and GBM6 cell lines were initially obtained from the Dr. Jann Sarkaria lab from the Patient-Derived Xenograft (PDX) National Resource at the Mayo clinic, who extensively characterized the genetic landscape and histological characteristics.

Human microglia cell lines: HMC3 and HPM cell lines

HMC3 is an immortalized microglia cell line isolated from the human brain of an embryo. HMC3 was purchased from and authenticated the American Type Culture Collection using genome sequencing (ATCC, catalog No. CRL-3304). HPM, or human primary microglia, used in this study were sourced from and molecularly authenticated by Celprogen (Catalog No. 37089-01). Originally, HPM cells were isolated by differential centrifugation from the postmortem human cerebral cortex. HPM were not immortalized and tested positive for a complement of extracellular matrix components specific to microglia including Mac-1, CD68, CD86, B7-2, HLA-ABC, HLA-DR, and RCA-1 lectin.

Murine GBM cells: TRP cell line

As mentioned above, TRP cells originate from genetically engineered C57/BL6 mouse GBm models.

Monocultures

All GBM38, GBM6, HMC3, and HPM cells were grown as adherent cultures in complete media made by supplementing Gibco Dulbecco's Modified Eagle Medium/ Nutrient Mixture F-12 (Ham) (DMEM/F-12, 1:1, 1X, Thermofisher, Catalog No. 11965-092) with 10% fetal bovine serum (FBS, Thermofisher, Catalog No. A5670701) and 1% penicillin/streptomycin (P/S, Thermofisher, Catalog No. 15140-122), unless otherwise mentioned. TRP cells were grown as neurospheres in serum-free neurosphere media made using DMEM-F12 media mentioned above supplemented with 20ng/ml animal-free recombinant human endothelial growth factor (EGF, Peprotech, Catalog No. AF-100-15), 20ng/ml recombinant human fibroblast growth factor- basic (FGF, Peprotech, Catalog No. 100-18b), Gibco B-27 supplement (50X) (Invitrogen, Catalog No. 17504-044), Gibco N-2 supplement (100X) (Invitrogen, Catalog No. 17502-048), 5% penstrep, and 1% Normamocin (Invitrogen, Catalog No. ant-nr-1). GBM38, GBM6 cells, HMC3 cells and HPM cells were grown as adherent cultures. Cultures were all maintained in a tissue culture incubator under controlled conditions (95% $O_2$, 5% $CO_2$, and 37°C).

**Targeted Metabolomics:**

Conditioned media metabolite sample processing:

We cultured equal number(250,000 of each cell type) of GBM cells and microglia as monocultures with 1.5mL media in 12-well plates, and microglia-GBM cocultures (contact) in a 50-50 ratio (250,000 of each cell type) with a total of 500,000 cells in 3ml media in 12 well plates, with each culture type in 6 replicates and ensuring supply of 1.5mL media per 250,000 cells. After 48 hours in culture, the conditioned media from each condition as well as control media were collected as 80% methanol solutions and maintained on dry ice during processing. The supernatant solutions were centrifuged three times at the maximum speed (approx.4000rpm) for 10 minutes at 4°C to pellet insoluble materials such as salts and proteins. The supernatants were collected and blow-dried using the nitrogen blower for approximately 2.5 hours to dry until

no liquid remained. Dried metabolite pellets resuspended in 50% methanol and subjected to LC-MS as described below.

Intracellular metabolite sample processing:

To study the transfer of NAG and NAA from microglia to GBM cells, the GBM cells flow sorted from cocultures as described above were lysed using 80% methanol and processed for metabolite extraction as discussed above.

To study the effect of NAG or NAA supplementation on the metabolic profile of GBM cells, we performed metabolic analysis of the cell extracts. 4-5 million cells were plated in 10cm dishes. 5 replicates received 100µM NAG or 1mM NAA. The replicates which did not receive NAG or NAA supplementation served as controls. One replicate for each condition was used for protein quantification for normalization during subsequent analysis. After 6 hours of incubation with or without NAG or NAA, the supernatant was removed and the cells were lysed using 80% methanol on dry ice. The cell lysates were then processed for metabolite extraction as discussed above.

**Stable Isotope Tracing:**

Experimental conditions

$^{15}$N-ammonium tracing: GBM cells cultured with 10mM $^{15}$N-labelled $NH_4$ (Cambridge Isotope Laboratories, Catalog No. NLM-467-1) in complete media with or without 100µM NAG supplementation (L-NH4+ NAG and L-$NH_4$ respectively were assessed for the effect of NAG supplementation on Urea cycle activity. Cells receiving 10mM unlabeled $NH_4$ (Millipore Sigma, Catalog No. A9434-500G) in complete media with or without 100µM NAG supplementation (UL-$NH_4$ +NAG and UL-$NH_4$ control respectively) served as tracing negative controls.

$^{13}C_5$ NAG tracing: GBM cells were cultured in complete media described above with $^{13}C_5$ NAG (treatment group) or no $^{13}C_5$ NAG (control group). Unlabeled control and unlabeled NAG supplemented cells served as tracing negative controls.

$^{13}C_4$ NAA tracing: GBM cells were cultured in complete media described above with $^{13}C_4$ NAA (treatment group) or no $^{13}C_4$ NAA (control group). Unlabeled control and unlabeled NAA supplemented cells served as tracing negative controls.

**Acetyl coA assay:**

To assess the effect of NAG or NAA supplementation on intracellular acetyl coa levels, we compared GBM cells cultured in complete media with GBM cells supplemented with 100uM NAG, 1mM NAA, 4M sodium acetate (Sigma, catalogue no. S2889) for 48hrs or subjected to overnight glucose starvation using Glucose free DMEM with 10% dialyzed FBS and 1% penstrep. The sodium acetate supplemented cells served as positive controls and the glucose starved condition served as negative control.

Intracellular acetyl-coA levels were assessed using a colorimetric assay (Novus Biologicals, NBP3-24455), after optimizing the manufacturer's protocol for GBM38 and GBM6 cells. The principle of this kit-based assay is illustrated here (supplementary methods, fig1.) Briefly, the total cell count was used for normalization instead of the wet weight of tissue, as mentioned in the protocol. At least 20million cells per condition were used for the assay. The cells were pelleted and snap frozen at the end of the treatment and processed for acetyl coA extraction the following day.  The extracted samples were then treated with the reaction mixture containing malate, NAD+, Malate dehydrogenase and citrate synthase enzymes. The NADH generated during the conversion of acetylcoA in the sample to citrate was measured as the change in measurement in 1 min at 340nm on Cytation 3 (Biotek) microplate reader.

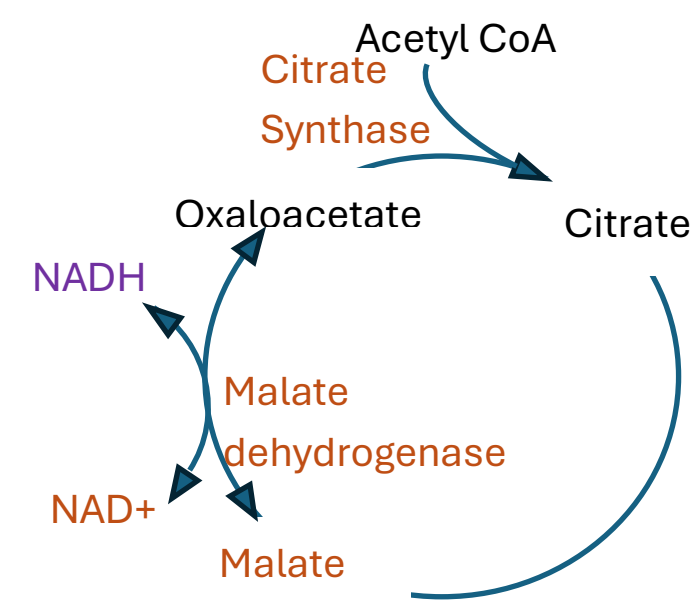


**Clonogenic Survival Assay:**

GBM cells were plated at clonal density. Control GBM cells, and GBM cells treated with 100µM NAG or 1mM NAA 10 minutes prior to irradiation, were irradiated at the indicated doses. The NAG or NAA treatment conditions were replenished every two days. After 14 days of incubation, colonies were stained and manually counted. Surviving fractions were tabulated across all conditions and plotted in order to compare radioresistance of NAG-supplemented cells to normal media-treated cells (GraphPad Prism, v.10.4.1). The radiation treatment enhancement ratio (ER) is the ratio of the mean inactivation dose in controls and the mean inactivation dose (Dmid) in NAG or NAA treated cells. Dmid is the area under the linear-quadratic survival curve for the condition. ER >1 signifies sensitization to RT, while <1 signifies resistance to RT.

Similarly, clonogenic assays were performed in GBM KAT5 knockdown cells to compare the radiation resistance conferred by NAG or NAA in the control cells (wildtype or sgControl) and the KAT5 knockdown cells.

**CRISPR/Cas9 Gene Knockdown and Transfection:**

To investigate the role of downstream metabolic pathways involved in NAG and NAA metabolism, CRISPR/Cas9-mediated gene knockout was performed as described previously[5]. Guide RNA (gRNA) constructs specific to the target gene, KAT5 (Edit-R Human Synthetic KAT5 (10524) sgRNA Set of 3, Horizon discovery, SQ-006301-01-0002) and  control gRNA (Horizon Discovery, cat noU-009501-01-02 )were transfected along with Cas9-mKate  mRNA (Horizon discovery, cat no. CAS11859) using Transfect Duo (Horizon discovery, cat no T-2010-01) according to the manufacturer's protocol.

Following transfection for 24hrs, cells were subjected to cell sorting using flow cytometry at the University of Michigan flow cytometry core to sort for mKate (a fluorophore which is included

as a reporter in the Cas9 mRNA) expressing cells. The cells thus obtained were further grown out into single cell-derived colonies and to generate stable knockdown colonies.

**Western Blot Analysis:**

To validate knockdown or knockout levels in CRISPR/Cas9, protein expression levels were assessed using standard immunoblotting techniques. Cells were lysed using RIPA buffer (Cat# 89900, Thermo Scientific) supplemented with protease inhibitor (Cat# 1187358001Roche) and phosphatase inhibitor (Cat# 04906845001, Roche). Protein was quantified using Pierce BCA assay. Primary antibodies against KAT5(a.k.a Tip60, Novus Biologicals, cat# NBP1-85482), AcetylH2AX ( Acetyl-Histone H2A.X (Lys5) Recombinant Rabbit Monoclonal Antibody (RM445),Themofischer, cat# MA5-44550) and β-Actin (Cat# sc-47778, Santa Cruz Biotechnology) were used. HRP-conjugated anti-rabbit (Cell signaling, 7074S) and anti-mouse (Cell signaling,7076S) secondary antibodies were used. The membranes were then treated with a chemiluminescent substrate (Brightstar ECL kit, Stellar Scientific, XR94). Membranes were developed using Bio-Rad ChemiDoc Imaging System.


1 Hao, Y. *et al.* Dictionary learning for integrative, multimodal and scalable single-cell analysis. *Nature biotechnology* **42**, 293-304 (2024).

2 Neftel, C. *et al.* An integrative model of cellular states, plasticity, and genetics for glioblastoma. *Cell* **178**, 835-849. e821 (2019).

3 Services, U. o. M. I. a. T. *Great Lakes High-Performance Computing Cluster*, <https://its.umich.edu/advanced-research-computing> (2026).

4 Zhang, S. *et al.* Elevated T Cell Immunoreceptor with Ig and ITIM Domains (TIGIT) Expression and Immune Cell Dysfunction Characterize Complex Proteins Associated With SET1 (COMPASS)—Like Complex Gene—Mutated Pancreatic Ductal Adenocarcinoma (PDAC). *Modern pathology : an official journal of the United States and Canadian Academy of Pathology, Inc* **39**, doi:10.1016/j.modpat.2026.100972 (2026 Feb 2).

5 W, Z. *et al.* GTP Signaling Links Metabolism, DNA Repair, and Responses to Genotoxic Stress - PubMed. *Cancer discovery* **14**, doi:10.1158/2159-8290.CD-23-0437 (01/12/2024).
